# Dynamic transformation of cubic copper catalysts during CO_2_ electroreduction and its impact on catalytic selectivity

**DOI:** 10.1038/s41467-021-26743-5

**Published:** 2021-11-18

**Authors:** Philipp Grosse, Aram Yoon, Clara Rettenmaier, Antonia Herzog, See Wee Chee, Beatriz Roldan Cuenya

**Affiliations:** Department of Interface Science, Fritz-Haber Institute of the Max Planck Society, 14195 Berlin, Germany

**Keywords:** Electrocatalysis, Electrocatalysis, Nanoparticles

## Abstract

To rationally design effective and stable catalysts for energy conversion applications, we need to understand how they transform under reaction conditions and reveal their underlying structure-property relationships. This is especially important for catalysts used in the electroreduction of carbon dioxide where product selectivity is sensitive to catalyst structure. Here, we present real-time electrochemical liquid cell transmission electron microscopy studies showing the restructuring of copper(I) oxide cubes during reaction. Fragmentation of the solid cubes, re-deposition of new nanoparticles, catalyst detachment and catalyst aggregation are observed as a function of the applied potential and time. Using cubes with different initial sizes and loading, we further correlate this dynamic morphology with the catalytic selectivity through time-resolved scanning electron microscopy measurements and product analysis. These comparative studies reveal the impact of nanoparticle re-deposition and detachment on the catalyst reactivity, and how the increased surface metal loading created by re-deposited nanoparticles can lead to enhanced C_2+_ selectivity and stability.

## Abstract (web summary)

Understanding the changes that occur in catalysts during reaction are key to the rational design. Here, the authors use electrochemical transmission electron microscopy and time-resolved product analysis to unveil how cubic copper oxide catalysts evolve during electrochemical CO_2_ reduction, linking their structural transformations with their selectivity.

## Introduction

The use of renewable energy to power the conversion of simple molecules, such as CO_2_ and H_2_O, into valuable products is a key strategy towards a sustainable society^1,2^. However, many of these technologies are not yet industrially viable because they are hampered by the lack of functional catalysts with good performance and stability. Among these processes, the electrochemical reduction of CO_2_ (CO_2_RR) is particularly appealing because it transforms a greenhouse gas into useful fuels and base chemicals, such as ethylene and ethanol at ambient temperature and pressure^3,4^.

Developing a catalyst with the optimal balance of activity, selectivity, and stability is, however, a non-trivial task. Although some catalysts may show better initial performance than others, they often lose their activities over time. Copper is the most attractive material for CO_2_RR because of its unique ability to generate C_2+_ products, but it suffers from poor selectivity^3–5^. Several studies have also suggested that the re-structuring of Cu-based nanostructures under applied potential can alter their catalytic properties^6–14^. Unfortunately, these conclusions are largely drawn from samples that have been removed from the electrochemical environment and evaluated in the absence of an applied potential. Detailed understanding of the specific morphological features responsible for a given activity and selectivity remains elusive due to insufficient insight into the dynamic evolution of Cu catalysts under reaction conditions^3,15,16^.

Liquid cell transmission electron microscopy (TEM) is a powerful technique for capturing the dynamics of nanostructures within a liquid environment^17–19^. Electrochemical experiments can also be performed within the TEM (EC-TEM) by incorporating thin-film electrodes on microfluidic chips^20^. Thus, the morphological evolution of electrocatalysts can be monitored under reaction conditions^13,21–27^ and, in combination with electrochemical measurements, used to establish structure-property relationships^18,28^. Such information is key to inform theoretical models for rational catalyst design and can provide the much-needed understanding of the parameters that influence the reaction pathways required for generating multi-carbon products^29,30^.

Here, we track the dynamic evolution of electrochemically synthesized Cu_2_O cubes in real-time under CO_2_RR conditions with EC-TEM and correlate the changes with the catalyst behavior obtained from benchtop electrochemical measurements. Our in situ observations revealed that the morphology and loading of the cubic pre-catalysts can change significantly in the electrolyte and under applied potential. We found nanoporous cubic frames and re-deposited nanoparticles (NPs) coexisting on the working electrode under reaction conditions, and that these re-structured catalyst particles can further aggregate or detach from the working electrode during extended reaction times. By tracking the reaction products generated by Cu_2_O cubes with different initial sizes and loadings for several hours in parallel to the extended in situ imaging of those cubes, we show how NP re-deposition and catalyst detachment impact the overall catalyst reactivity. In particular, our results indicate that a dense working catalyst network made up of stable nanoporous cubes and re-deposited NPs can sustain high CO_2_RR selectivity towards C_2+_ products.

## Results

**Fig. 1:**
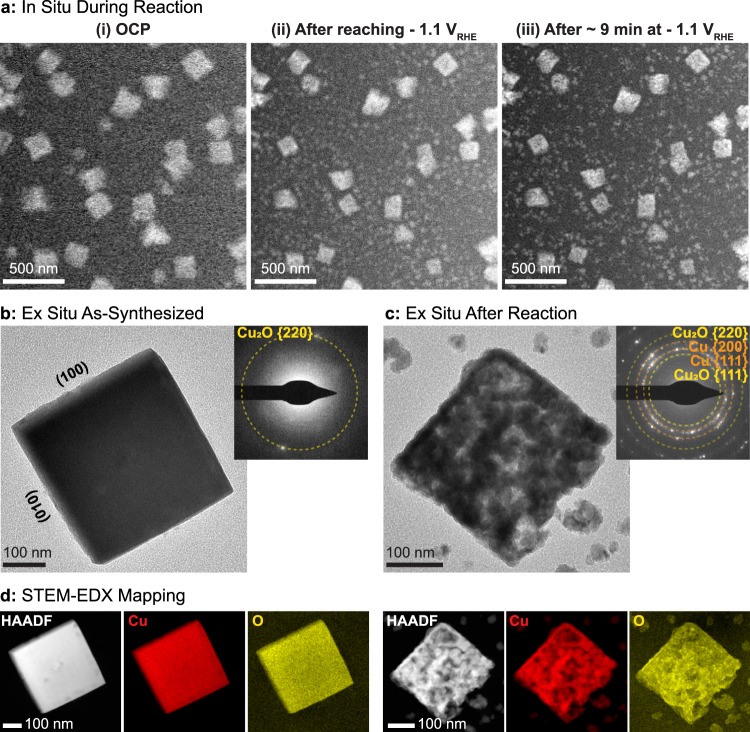
Morphology of Cu_2_O cubes and their evolution during CO_2_RR. **a** Sequence of images illustrating the morphological changes observed in ~170 nm cubes after (i) introducing CO_2_ saturated 0.1 M KHCO_3_ under open circuit potential, (ii) while applying a reductive potential of −1.1 V_RHE_ and (iii) after ~9 min at −1.1 V_RHE_, where a large bubble formed. The image sequences were all acquired with an electron flux of 1.7 e^−^ Å^−2^ s^−1^. Comparison of Cu_2_O cubes **b** before and **c** after reaction using ex situ TEM imaging and electron diffraction (upper right inserts). **d** STEM-EDX maps of the same cubes showing the Cu and O signals.

In the EC-TEM studies, we followed the morphological evolution of Cu_2_O cubes with three size distributions, 80 ± 5, 170 ± 40, and 390 ± 60 nm. The cubes were synthesized directly on the carbon working electrode of an EC-TEM chip ex situ from an aqueous mixture of CuSO_4_ and KCl^31^ in a standard benchtop electrochemical setup (Supplementary Fig. 1). With this synthesis protocol based on electrodeposition, we were able to achieve reproducible size and loading of the pre-catalyst cubes (Supplementary Table 1). In Fig. 1a, we show a scanning transmission electron microscopy (STEM) image sequence where we imaged several ~170 nm Cu_2_O cubes in situ under different electrochemical conditions, (i) at open circuit potential in CO_2_-saturated 0.1 M KHCO_3_, (ii) after applying −1.1 V_RHE_, and (iii) after ~9 min at −1.1 V_RHE_, where a bubble formed. The image sequence shows that a few morphological changes occur during the application of the reductive potential: new NPs (~40 nm) form, and the cubes become porous and decrease in size by 10%. The size decrease can be attributed to the reduction of Cu_2_O towards metallic Cu as previously determined from operando spectroscopy studies^10,32,33^. The re-deposited NPs form just below −0.3 V_RHE_, before the size reduction of the cubes takes place at about −0.7 V_RHE_ (Supplementary Fig. 2). This suggests that the small NPs are not produced by the reduction of Cu_2_O, but originate from Cu species leached from the cubes after they were immersed in the electrolyte^10,27^. In addition, due to the nature of these in situ experiments, the re-deposited NPs must be forming on the carbon electrode^34^. The high-resolution image in Fig. 1a(iii) acquired after bubble formation also reveals more clearly the nanoporous nature of the cubes. A comparison of Fig. 1a(ii) and (iii) further shows that the nanoporous cubes remained stable under sustained applied potential, whereas the small NPs started to agglomerate into interconnected structures. We note here that two initial linear sweeps are always taken during these experiments, the first to check for day-to-day shifts in the reference potential and the second for confirmation after correcting any identified shifts (Supplementary Note 1). Representative examples of the movies recorded during different experiments as the potential was changed from open circuit potential to −1.1 V_RHE_ during the 1st and 2nd linear sweeps are presented as Supplementary Movies 1 and 2, respectively. The images in Fig. 1 are extracted from the experiments shown in Supplementary Movie 2. We also present in Supplementary Fig. 3 the wider field-of-view images acquired before and after the linear sweeps to −1.1 V_RHE_ to indicate that the re-structuring of the cubes and the re-deposition of NPs extend beyond the area imaged during the potential sweep, which confirms that these changes are not artifacts of the electron beam irradiation. The subsequent image sequences recorded during sustained reaction at −1.1 V_RHE_ are provided as Supplementary Movie 3.

Figure 1b–d shows ex situ TEM data, selected area electron diffraction and STEM-energy-dispersive X-ray spectroscopy (EDX) maps of a ~390 nm Cu_2_O cube acquired (b) before and (c) after CO_2_RR at −1.1 V_RHE_ for 1 h in CO_2_-saturated 0.1 M KHCO_3_. In agreement with the image sequence in Fig. 1a, the single crystalline Cu_2_O cube is fragmented and porous after the reaction. EDX spectra acquired from the cubes (Supplementary Fig. 4a) indicate a significant decrease in both O and Cl signatures in these Cl^−^-stabilized Cu_2_O cubes after the reaction. The electron diffraction pattern exhibits strong Cu and weak Cu_2_O diffraction spots after reaction, where the Cu_2_O signatures may be due to re-oxidation during the ex situ sample transfer. The smaller NP agglomerates that are formed during the reaction were identified as Cu (Supplemental Fig. 4b).

Bubble formation at −1.1 V_RHE_, however, poses a problem for our in situ investigations. The smaller cubes (80 nm) and the re-deposited NPs can be swept away by the passage of a gas bubble (see for example Supplementary Fig. 5 and Supplementary Movie 4), which prevent us from continuing our observations. As it was challenging to prevent the formation of gas bubbles and to remove them at −1.1 V_RHE_, we applied a lower potential of −0.9 V_RHE_ in the subsequent experiments, which allowed us to extend the continuous observation window up to about an hour. This potential was chosen because the formation of multi-carbon products, such as C_2_H_4_, from the Cu_2_O cubes is already expected at approximately −0.9 V_RHE_^10^. A comparison of the image sequences of cubes with similar sizes collected at −0.9 V_RHE_ and −1.1 V_RHE_ indicates that there was increased catalyst detachment and aggregation at the more negative potentials, but no noticeable differences in the catalyst morphology, which implies that the more negative potential only accelerated the morphological changes.

**Fig. 2:**
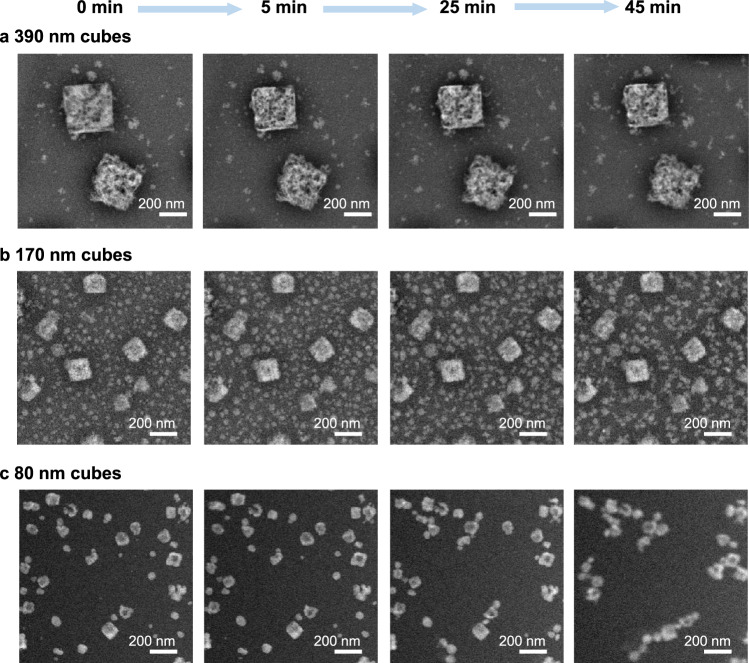
Morphological evolution of Cu_2_O cubes and re-deposited NPs captured under operando conditions over 45 min of CO_2_RR by EC-TEM. Snapshots extracted from the videos tracking the evolution of **a** 390 nm, **b** 170 nm, and **c** 80 nm cubes at 0, 5, 25, and 45 min after applying a potential of −0.9 V_RHE_. Each image is an average of 10 frames. The electron flux used is 3.5 e^−^ Å^−2^ s^−1^.

Figure 2 depicts the morphological evolution of cubic-shaped Cu_2_O catalysts at −0.9 V_RHE_ with different initial sizes where the image sequences track the same cubes at four different reaction times after the application of the reductive potential: *t* = 0, 5, 25, and 45 min. The corresponding movies with wider fields-of-view for the size distributions of (a) 390 nm, (b) 170 nm, and (c) 80 nm are provided as Supplementary Movies 5, 6, and 7, respectively. The representative examples of the currents measured during these chronoamperometric experiments are plotted as Supplementary Fig. 6. A comparison of the image sequences shows a drastic difference between the larger (390 and 170 nm) and the smaller (80 nm) cubes. Although fragmentation and a size decrease were found to occur under reductive potential regardless of the initial cube size, the cubic nanoporous frames from larger cubes were largely stable and did not show significant mobility. On the other hand, the 80 nm cubes decreased in number after the introduction of electrolyte. Different areas of the working electrode showed either a noticeably lower cube loading or remnant cube fragments (compare with Supplementary Table 1). To confirm this observation, we imaged the Cu_2_O cubes ex situ with TEM, after synthesis, after a 30-min immersion in 0.1 M KHCO_3_ and after reaction at −0.9 V_RHE_ in our standard H-type cell (Supplementary Fig. 7). While large cubes (≥170 nm) only showed subtle changes after immersion in the bicarbonate solution, the smaller cubes underwent significant degradation and a reduction in their number, corroborating the poorer stability of these cubes. The dynamics of the 80 nm cubes under applied potential, as described by the in situ TEM image sequence in Fig. 2c, also indicate further catalyst aggregation and detachment under reaction conditions. Additional EC-TEM experiments, performed using cubes with the same three size distributions shown above, depict behaviors consistent with that described in Fig. 2 (Supplementary Figs. 8, 9 and Supplementary Movies 8–11).

### Catalytic performance of similarly synthesized cubes

**Fig. 3:**
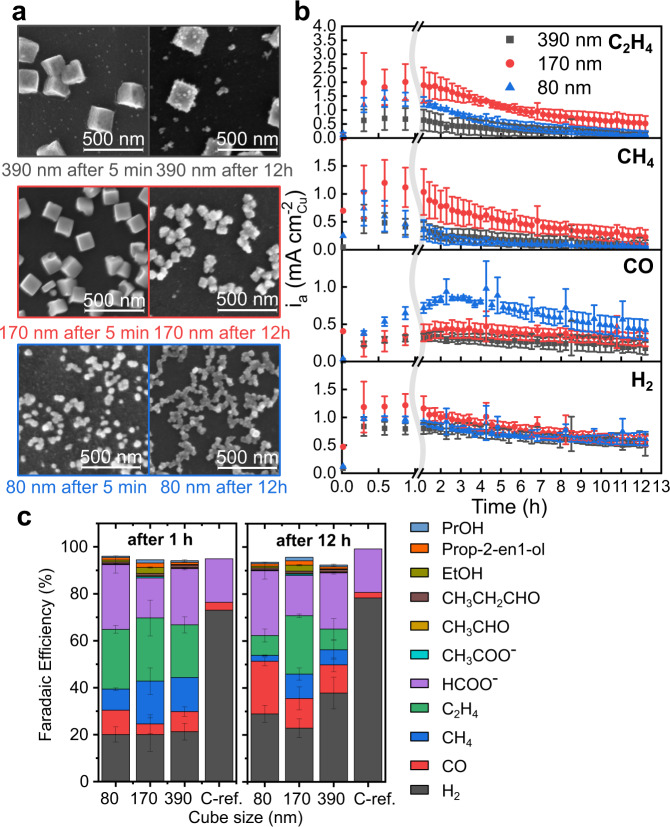
Product analysis from CO_2_RR over Cu_2_O cubes deposited on glassy carbon. **a** SEM images of Cu cubes with three different average size distributions measured ex situ after 5 min and 12 h of CO_2_RR. **b** Partial current densities for H_2_, CO, CH_4_, and C_2_H_4_ obtained from time-resolved gas chromatography data acquired over 12 h. The currents are normalized by the exposed surface area of the as-synthesized Cu_2_O cubes. Note that this normalization does not consider the dynamic morphological changes observed under reaction conditions. Faradaic efficiencies of all measured reaction products after **c** 1 h and 12 h of CO_2_RR at −1.1 V_RHE_. The efficiency in **c** is slightly lower than 100% due to evaporation of liquid products and presumably non-linear product formation, since we average the liquid products over 12 h. The reference sample is a glassy carbon plate without catalysts. The error bars give the standard deviation of three measurements.

To understand how the morphological changes identified in the in situ experiments impact the overall catalytic properties of these samples, we repeated the synthesis of the Cu_2_O cubes on conventional glassy carbon plates using the same protocol and measured their reaction products using online gas chromatography. The cubes synthesized on the EC-TEM chips and the glassy carbon plate are similar in morphology, with only minor differences in size and loading. The size and distribution of cubes on the chips and the glassy carbon plates are reported in Supplementary Table 1 and comparative SEM images in Supplementary Fig. 10. Chronoamperometric traces obtained during these experiments are provided in Supplementary Fig. 11. Results from online gas chromatography analysis of these samples, measured at −1.1 V_RHE_, are shown in Fig. 3. Here, Fig. 3a shows SEM images of the samples after 5 min and 12 h of CO_2_RR and Fig. 3b plots the partial current densities for hydrogen (H_2_), carbon monoxide (CO), methane (CH_4_), and ethylene (C_2_H_4_) for samples with different initial size distributions. It can be seen from Fig. 3a that the cubic structure was preserved for the larger cubes, but with a roughened surface, whereas extensive agglomeration was already observed for the smallest ones after 5 min, consistent with the in situ EC-TEM results. The relatively small amounts of re-deposited NPs seen after 12 h also suggest that most of them had either attached onto the existing cubes or detached from the carbon support during the reaction. As mentioned earlier, we only expect the more negative potentials to accelerate the dynamic changes in the morphology but they should not change the underlying structural characteristics. A comparison of the sample morphologies reacted at −0.9 V_RHE_, −1.1 V_RHE_, and −1.3 V_RHE_, captured using SEM (Supplementary Fig. 12a) shows that the general morphological evolution is comparable among the different potentials and supports our view about the relation of the morphologies with the applied potentials. Partial current densities for gaseous products at −0.9 V_RHE_ and −1.3 V_RHE_ are also provided as Supplementary Fig. 12b.

It can be seen from Fig. 3b that over the course of 12 h at −1.1 V_RHE_, CH_4_ and C_2_H_4_ production decrease, which is explained by the observed degradation of the catalyst cubic morphologies, especially with the attachment of re-deposited NPs onto the fragmented cubes. The details, however, differ across the three samples. The sample composed of 170 nm cubes showed significantly higher currents for CH_4_ and C_2_H_4_ compared to the other two samples, presumably due to the higher density of catalytic structures present on the working electrode surface in this sample as shown in Fig. 2b. On the other hand, the 80 nm cubes showed a precipitous drop in partial current density for CH_4_ and C_2_H_4_, and an increase in the partial current density for CO within the first hours, which we attribute to the detachment of the catalysts from the working electrode surface as seen in the in situ experiments. While the yields of H_2_ and CO stayed roughly constant for the 390 and 170 nm cubes, they reached a similar level for the three samples after the first hour of reaction. The 390 nm cubes also maintained the selectivity towards CH_4_ and C_2_H_4_ that were close to those originally found in the 80 nm cubes, but with a more gradual drop over time.

Figure 3c further compares the Faradaic efficiencies (FE) towards different products at 1 and 12 h of reaction time for these three samples. Supplementary Fig. 13 provides the full data sets of the FEs for all products over 12 h with a temporal resolution of about 17 min. Supplementary Fig. 14 compares the gaseous products in terms of the FE for the different cube sizes. It is evident from the product analysis that the 170 nm cubes were the only ones that retained significant hydrocarbon selectivity after 12 h.

We further performed experiments where identically prepared cubes on glassy carbon were extracted from a beaker and examined in SEM at regular intervals during the first hour of the CO_2_RR to track the time-resolved morphology changes of the cubes in the H-type cell. These ex situ imaging experiments are summarized in Supplementary Note 2. The main features of the EC-TEM observations; cube fragmentation, re-deposition of small NPs, and their subsequent agglomeration were found in these samples, and their trends as a function of the cube size were also reproduced, indicating that our EC-TEM experiments indeed mimic the behavior of the electrocatalysts in benchtop systems.

### Describing the dynamic evolution of Cu_2_O cubes

**Fig. 4:**
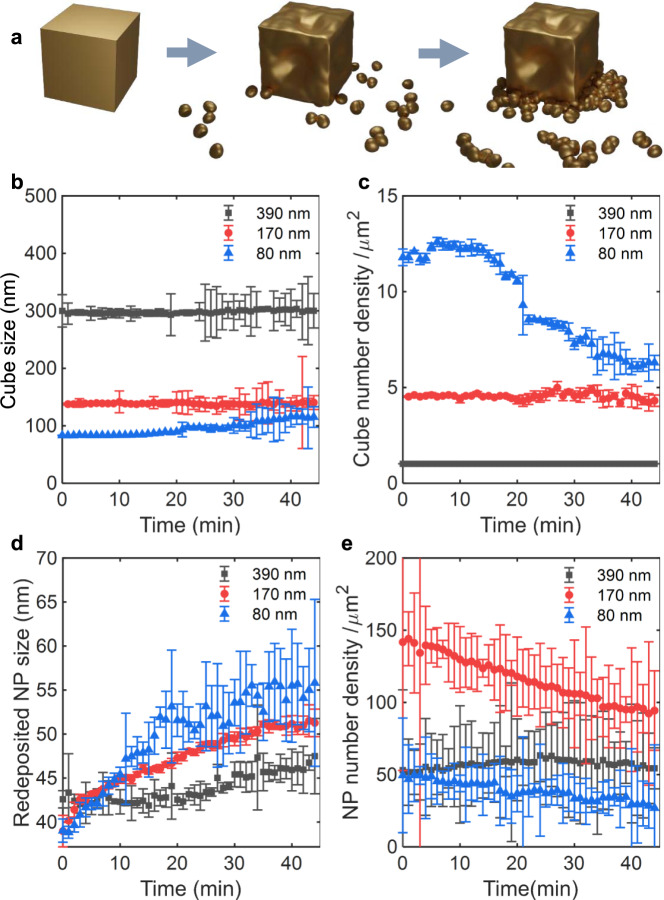
Catalyst size changes over 45 min from in situ imaging. **a** Schematics describing the evolution of the cube morphology and the re-deposition of catalyst particles. **b** The average cube size, **c** the number density of cubes, and **d** re-deposited particle size and **e** the number density of the re-deposited particles extracted from in situ CO_2_RR experiments collected over 45 min. The error bars represent the segmentation error from the automated NP detection processing routine (see “Methods” section for details).

Hence, the formation, motion, and aggregation of the re-deposited NPs are evidently the main drivers of the morphological change on the working electrode surface. Figure 4 plots the time-resolved traces of the size of the cubes and re-deposited NPs extracted from the EC-TEM studies. After the reductive potential is applied, the size of the cubes decreased by 10~15% from their as-synthesized size as seen in Fig. 4b. Following this initial size change, the cubes remained stable in size but with an increasing spread in sizes as the small re-deposited NPs start to attach to the corners and edges of the cubes as presented in Fig. 4b. The cubes with an initial size of ~80 nm showed a steep increase of their size as compared to the larger ones because these small cubes became more mobile, which leads to significant aggregation during sustained potential application. As can be seen in Fig. 4c, the number density of 390 and 170 nm cubes stay constant during the first hour, whereas the number density of 80 nm cubes decreases by ~50%. The latter is explained by the higher mobility, extended aggregation, and detachment of the smallest cubes, which originate from their poor adhesion to the working electrode surface, as shown in Supplementary Fig. 7.

As for the re-deposited NPs, their initial sizes were similar at ~40 nm for the three sample sets as seen in Fig. 4d. As these small NPs aggregated with each other or attached to the surface of the existing cubes over time, their size further increased from 40 to 60 nm. The NP number density also decreased by ~25% over 45 min for the 170 and 80 nm cube samples as shown in Fig. 4e. Such aggregation behavior seems to be similar to that reported for small Cu NP ensembles during CO_2_RR^6,13^. The NPs in the 390 nm cube sample show only a minor change in NP surface density, probably due to the sparse distribution of NPs in the original as-prepared sample, Fig. 2a, which limits NP aggregation.

### Correlating morphology with catalytic performance

These changes in the catalyst morphology can be further matched with the  overall trends in the activities of the differently sized cubes. The smallest cubes studied were clearly the least stable. Significant aggregation and loss of catalyst material from the glassy carbon surface were observed during the reaction, which leads to a drastic drop in the density of nanocubes in the 80 nm sample. The latter is further reflected in the sharp decrease in their hydrocarbon production. Small Cu NPs are also known to be selective towards CO and H_2_, instead of hydrocarbons^35^. On the other hand, the increased selectivity towards C_2+_ products in the 170 nm cube sample is surprising and requires us to consider how the catalyst loading changes under reaction conditions, since this is expected to impact its selectivity^36,37^. In the as-synthesized sample, the highest catalyst loading was obtained for the 80 nm cubes and the lowest for the 390 nm cubes^31^. However, the EC-TEM observations indicate that during CO_2_RR, the number density of both, cubes and NPs formed during the reaction, is three times higher in the 170 nm sample as compared to the other two samples. Therefore, the high coverage of the NPs generated during CO_2_RR from the 170 nm cubes is most likely responsible for the higher amount of multi-carbon products (e.g., C_2_H_4_) produced. A more facile C-C coupling, leading to enhanced hydrocarbon selectivity (vs CO) was previously reported for Cu CO_2_RR electrocatalysts as a function of the interparticle distance^36^ and Cu mass loading^37^. Moreover, the fragmentation of the as-synthesized Cu_2_O cubes intrinsically creates a large number of defects (e.g., edge sites), which have been deemed favorable for enhanced CO_2_RR catalytic activity and hydrocarbon product selectivity^15,38^. The exact effect of such defects, however, remains to be clarified, as very small NPs (<5 nm) with a large number of undercoordinated sites have also been shown to favor H_2_ formation^35^.

Interestingly, the 170 nm cubes also had the most stable catalytic performance. FE towards C_2+_ products only dropped by 24% for the 170 nm sample over 12 h, as compared to 46% for the 390 nm sample and 54% for the 80 nm sample. Previously, Jung et al.^12^ had reported that 20 nm Cu_2_O nanocubes synthesized by means of wet chemistry can gradually fragment over several hours, resulting in a corresponding increase in ethylene selectivity. In comparison, our larger (80–390 nm) electrochemically-grown cubes fragment almost instantaneously upon the application of a reductive potential, attain peak current densities for C_2+_ products within the first hour of reaction as shown in Fig. 3 (and Supplementary Figs. 13 and 14), but only the 170 nm cubes sustain their selectivity. This long-term catalytic stability may be explained as a combination of the morphological stability of the larger nanoporous cubic frames during sustained potential application and the formation of densely packed re-deposited NPs aggregates around the cubes, giving rise to a reduction in interparticle spacing during reaction^36^.

### Effect of adjusting the cube loading at different sizes

**Fig. 5:**
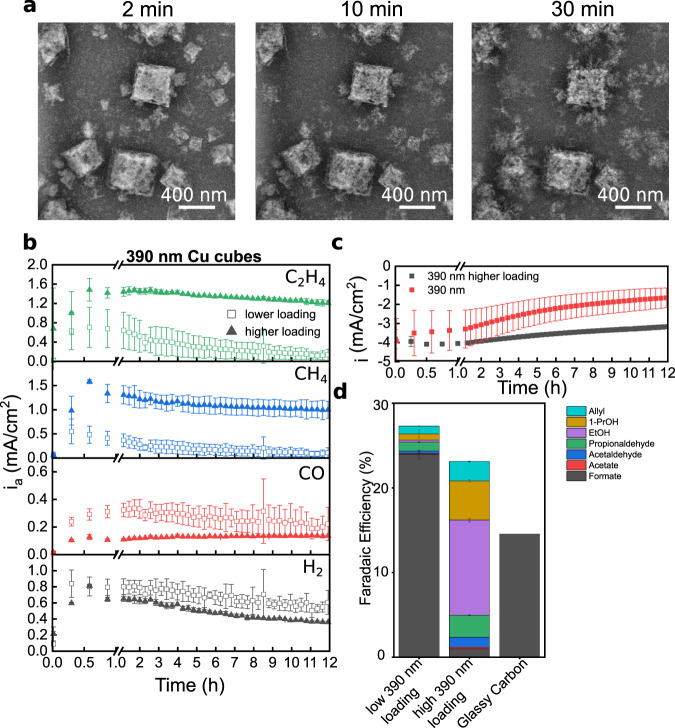
Comparison of the Cu cube behavior at different loadings. We compare nominally 390 nm Cu cubes at high loading (**a**) at the same potential. In **b**, we compare the partial current density for the gas products of the high loading sample (390 nm Cu cube size) normalized by the initial loading. We have observed a higher overall current (**c**) and more C_2+_ gas and liquid products (**b**, **d**) at higher loading at −1.1 V_RHE_. The error bars give the standard deviation of three measurements.

To verify these trends, we repeated these experiments with samples for which the synthesis protocols were adjusted to increase the loading of the 390 nm cubes (~300% more, from 9 ± 2% to 27 ± 5%) and decrease the loading of the 170 nm cubes (~25% less loading, from 26 ± 5% to 19 ± 2%). See Supplementary Movies 12 and 13, respectively. The results for the 390 nm cubes are summarized in Fig. 5. Although the catalyst sizes were not exactly reproduced, it was clear that a packed surface morphology made up of nanoporous cubes and aggregated re-deposited NPs was seen again when the loading of the as-synthesized large cubes was increased. More importantly, the partial current density for the 390 nm samples with increased loading clearly indicates a higher selectivity for C_2+_ hydrocarbons (gas products and liquid products) as well as lower amounts of CO, H_2_, and formate as shown in Fig. 5b, d. Time-resolved product measurements are also presented in Supplementary Fig. 15. We attribute this trend to the higher density of re-deposited Cu NPs and the resulting interconnected network of Cu particles that emerges during the first hour(s) of applied potential. As can be seen in Fig. 5c, the total current density normalized over the initial loading also displays higher stability over 12 h at higher loadings. Decreasing the loading of the 80 nm cubes, on other hand, leads to a significant drop in hydrocarbon formation (Supplementary Fig. 16), which is consistent with our presented findings. Since these results also demonstrate that the concomitant dynamical morphology of cubes and re-deposited NPs is needed to ensure high catalytic selectivity and stability, it mitigates any concerns regarding the impact that the higher loading found in the glassy carbon samples could have on our conclusions (Supplementary Fig. 9).

Here, we also address a common concern with in situ electron microscopy experiments, namely, artifacts caused by radiolytic products generated by the electron beam^28,39–41^. In Supplementary Note 2, we discuss the control experiments we performed to rule out the electron beam-induced effects in these extended imaging experiments. Our control experiments indicate that the electron beam did not alter the structural characteristics of the catalyst. Nevertheless, there can be chemical effects from electron beam irradiation, which we will briefly discuss here. In pure water, electron irradiation is generally expected to create reducing radicals and shift the pH towards lower values^39,40,42^. Bicarbonate-containing aqueous solutions, however, are expected to result in net oxidizing conditions under irradiation^42^, which will lead to particle dissolution in our samples rather than the more commonly reported beam-induced particle deposition. This is consistent with our observations of gradual cube dissolution at electron fluxes above 7 e^−^ Å^−2^ s^−1^. On other hand, it is not yet understood how bicarbonate species alter possible pH shifts resulting from the electron beam. According to the Pourbaix diagram for Cu in water^43^, Cu_2_O is only stable above pH 6 (unsaturated 0.1 M KHCO_3_ has a pH of 8.3). Since the cubes were stable under our imaging conditions, it was unlikely that the pH was shifted drastically below the pH of 6.8 for CO_2_-saturated KHCO_3_.

## Discussion

In short, we have presented a realistic picture of how Cu_2_O catalysts dynamically re-structure under CO_2_RR reaction conditions using both in situ and ex situ electron microscopy, providing insight into the kinetics of different dynamical processes such as re-deposition, agglomeration, detachment, dissolution, and fragmentation^16^. Clearly, the catalytic properties of our system cannot be interpreted using the concepts based on the flat surfaces of the well-defined as-prepared Cu_2_O cubes. Instead, the catalytic reactivity is intricately linked to the catalyst stability and the dynamic loading/particle distribution under reaction conditions. For example, the catalytic stability of the cubic Cu_2_O particles could not be easily understood if one simply considered a size dependence, because neither the samples with sparse, larger, and stable cubes nor the samples with smaller, rapidly aggregating NPs showed the best performance. Instead, a combination of densely loaded cubes and a high density of re-deposited NPs yielded the best C_2+_ product selectivity and stability, alluding to a complex interplay between the initial catalyst parameters (cube size, cube loading) and their dynamic parameters (nanoporous frame stability, re-deposited NP size, re-deposited NP loading, re-deposited NP aggregation, and detachment). In addition, our results reveal that the detachment of the smaller catalyst particles and re-deposited NPs can play a major role in the degradation of the catalyst performance, which is difficult to probe through ex situ collected images. It is clear from the observed motion and aggregation of these catalysts that they are only weakly bound to the electrode surface, and therefore, they might become detached and lost when the sample is removed from the electrolyte and rinsed. To check the generality of our results, we also performed EC-TEM experiments using ~30 nm Cu_2_O cubes synthesized via a colloidal chemistry approach (Supplementary Fig. 17 and Supplementary Movie 14). Similar re-structuring and re-deposition were seen in these samples. Like our electrochemically synthesized ~80 nm cubes, these small cubes did also not attach well to the working electrode and were mobile during the extended reaction times employed, allowing us to extend our conclusions towards smaller catalyst sizes.

Furthermore, we showed that the number of re-deposited NPs is determined by the combination of the initial cube loading and interparticle distance as well as by the initial cube size. Thus, our EC-TEM observations, where the majority of re-deposited Cu NPs form during the initial ramp towards the reductive potentials, indicate that the material stems from the dissolution of Cu_2_O and that most of the leached Cu species are not lost to the electrolyte. This is corroborated by experiments conducted at higher flow rates, where no significant difference in the formation of re-deposited NPs is observed at four times the initial flow rate (Supplementary Fig. 18). In addition, identical location SEM images comparing the larger cubes after 15 h of being immersed in 0.1 M KHCO_3_ in the absence of the applied potential indicate no significant reduction in cube size, which suggests that the dissolution is only limited to the cube surface and initial electrolyte exposure (Supplementary Fig. 19). Subsequently, the application of the more negative potential under working conditions leads to the reduction of Cu_2_O to metallic Cu, and no further dissolution/re-deposition is seen. The ensuing fragmentation of the cubes can be explained by the rapid reduction of Cu_2_O to Cu preventing the cubes from attaining their thermodynamically favored structure with minimal exposed surface area.

We also reiterate here that the observed morphological transformations can be rationalized based on the redox chemistry of Cu. Previously, we had reported that the simple introduction of the Cl-stabilized CuO_x_Cl_y_ cubes into the electrolyte at open circuit potential can lead to Cl leaching from the cubes^10^. However, we do not expect the leached Cl^−^ to have a significant impact on the catalytic properties because of its extremely low concentration in the current work and Cl^−^ having the weakest effect among the halides that we previously considered^44^. It is also known from other studies, that Cu_2_O dissolves partially and Cu can re-deposit when we go from OCP towards CO_2_RR conditions^27,43^. These dissolution effects combined cause the decreased stability of the cubes with decreasing size, which gave rise to the observed changes in the catalytic selectivity.

Lastly, we emphasize that the complex evolution of the catalyst morphology and loading we have described will be inherent in any CO_2_RR system involving similar materials. In particular, our results with well-controlled pre-catalyst morphologies revealed that the loading of the pre-catalysts is likely an unreliable indicator for the loading of the actual catalyst during CO_2_RR, since the number of catalytically active particles can be reduced via detachment or increased via re-deposition. Therefore, any interpretation of the selectivity trends must take into consideration the morphology that exists under reaction conditions and be rationalized based on the contributions of both, Cu_2_O cubes and re-deposited NPs (summarized in the cartoon in Fig. 4a), and the changes in catalyst active area caused by the restructuring that occurs under reaction conditions. To extrapolate the effect of the catalyst size on catalytic selectivity, it is not enough to solely determine the size and the loading of the pre-catalysts. Instead, the working morphology and spatial distribution of the catalysts under reaction conditions is also needed. Since the only methods to obtain such information reliably are currently based on in situ microscopy, our results highlight a critical need for new techniques that can measure the electrochemical surface area^5^ in a minimally invasive and time-resolved manner. Further clarification using theory regarding how different factors (e.g., the effect of confinement in nanopores^45^) influence the catalytic behavior can further inform rational strategies that exploit the dynamic morphologies of these catalysts for improved performance.

In summary, using a combination of in situ and ex situ electron microscopy experiments, we showed that cubic Cu_2_O catalysts re-structure from a solid single crystalline form to a fragmented nanoporous structure under CO_2_RR conditions. Furthermore, we observed the formation of small randomly shaped NPs originating from Cu leached out of the Cu_2_O cubes, resulting in a dynamic morphology with two distinct structures: nanoporous cubic frames and re-deposited NPs. By matching the morphological evolution of the cubic catalysts and the smaller NPs generated by the applied potential with the analysis of reaction products from identically prepared samples deposited on bulk carbon electrodes, we elucidate how the C_2+_ hydrocarbon selectivity is controlled by initial cube size and surface coverage. In particular, we reveal that a dynamically evolving surface morphology consisting of nanoporous structures created from larger cubes intermixed with re-deposited NP aggregates can sustain catalytic selectivity towards hydrocarbons. Our results also demonstrate the inherent complexity of real-world electrocatalysts and their dynamic morphology under reaction conditions. Even when we start from well-controlled pre-catalyst samples with simple initial morphologies, significant changes in morphology and loading can result from catalyst detachment, fragmentation, and re-deposition processes, and an understanding of several structural parameters is still needed to rationalize the catalytic selectivity. Therefore, a complex interplay of dynamic structural behavior, dynamic loading, and interface stability must be considered for the development of better CO_2_RR catalysts. Gaining such insight into the long-term stability of nanoscale catalysts is especially important for the advancement of energy conversion technologies as it can lead to the development of novel strategies that make use of these inherent re-structuring dynamics to create catalysts with improved performance.

## Methods

### Cubic Cu_2_O synthesis

The synthesis protocol involves a mixture of 5 mM copper sulfate-pentahydrate (CuSO_4_·5H_2_O, Sigma Aldrich) and different concentrations of potassium chloride (KCl, Sigma Aldrich)^10,26^. Polished glassy carbon (vitreous, SPI) plates, as well as Hummingbird EC-TEM chips, were used as substrate. The largest Cu_2_O cubes of 390 nm are obtained using 5 mM KCl and the smaller cubes of 170 and 80 nm by increasing the KCl concentration to 30 and 50 mM, respectively^31^. The starting cubes were synthesized by electrochemical cycling between an oxidizing (+0.6 V vs. RHE) and a reducing potential (+0.1 V vs. RHE) for total 10 cycles, which led to the electrodeposition of size- and shape-controlled Cu cubes with a narrow size distribution depending on the Cu to Cl ratio used in the precursor. Initially, a potential of 0.6 V_RHE_ was held for 8 s with a subsequent potential jump to 0.1 V_RHE_ for 4 s. Returning to the initial potential completes the protocol. After the synthesis, the samples were rinsed with ultrapure water and then used for the subsequent CO_2_RR experiments.

### Electrolyte (KHCO_3_) preparation

All electrochemical experiments for CO_2_RR were performed in a 0.1 M KHCO_3_ solution as the electrolyte. The solutions were prepared by dissolving potassium bicarbonate (KHCO_3_, Sigma Aldrich) in ultrapure water (18 MΩ cm^−1^, from Veolia Purelab Flex Pure Water). Before use, purification of the solution with Chelex 100 resin (Bio-Rad) was performed for 24 h. Immediately prior to the measurement, the electrolyte was saturated with CO_2_ for at least 45 min to achieve full saturation.

### Ex situ electrochemistry and product analysis

Ex situ electrochemistry and product analysis measurements were carried out in a benchtop electrochemical setup using an Autolab potentiostat (PGSTAT 32N). An H-type two-compartment electrochemical cell with anolyte and catholyte separated by an ion-exchange membrane (Selemion AMV, AGC Inc.) was used with a Pt-mesh counter electrode (MaTecK, 3600 mesh cm^−2^), a leak-free Ag/AgCl (sat.) reference electrode (Innovative Instruments Inc.) in a three-electrode configuration. The working electrode compartment was stirred at 800 RPM. For each data point, at least three identical samples were measured.

The volatile gas products were analyzed via online gas chromatography (GC, Agilent 7890A) equipped with a thermal conductivity (TCD) and a flame ionization (FID) detector. Here, the headspace of the cell was directly connected to the GC, allowing online analysis after ~30 min of product accumulation. An injection was performed approximately every 17 min during the measurements. A continuous supply of CO_2_ was ensured by constant bubbling of the anolyte and catholyte with CO_2_ at 20 mL min^−^^1^. Carboxylates such as formate and acetate were measured after the experiment by high-performance liquid chromatography (HPLC, Shimadzu Prominence) equipped with a NUCLEAOGEL SUGAR 810 column and a refractive index detector (RID). Alcohols and other liquid products were analyzed by liquid gas chromatography (L-GC, Shimadzu GC-2010 Plus with auto sampler) equipped with a TCD and FID. We mention here that the first 1–2 injections at the beginning of the experiments tend to be less accurate due to limited product accumulation. All measurements were repeated three times on identically prepared and independent samples for statistical significance. The formulas used for these calculations and for obtaining the RHE potential are shown in Supplementary Note 3.

### In situ transmission electron microscopy

The in situ EC-TEM experiments were performed in a ThermoFisher 300 kV Titan TEM (ThermoFisher Scientific) operated in STEM mode using a Hummingbird Scientific Generation V Bulk Liquid Electrochemistry TEM holder (Hummingbird Scientific) with Pt counter and Ag/AgCl (3 M KCl) reference electrodes. A schematic of the experimental configuration can be found in Supplementary Fig. 20. The image sequences were acquired using an electron probe current of ~220 pA and at a frame rate of 1 frame per second with 1024 × 1024 pixel image resolution. The EC-TEM chips with a 50 nm thick silicon nitride membrane window were also produced by Hummingbird Scientific. The EC-TEM chips have a carbon film covering approximately half of the working electrode window. The bottom chips also have a 50 nm silicon window and 250 nm spacers. The electrochemistry experiments were performed using a Biologic SP-200 potentiostat. The potentials were measured against the built-in Ag/AgCl reference and then converted to RHE. Supplementary Note 1 also discusses the electrochemical performance of the EC-TEM holder as compared to a standard H-type cell and our procedure for calibrating the applied potentials between two systems.

The TEM holder was pre-filled with 0.1 M KHCO_3_ during cell assembly to ensure that the electrolyte fills the entire fluid path. After loading into the TEM, the syringe was filled with freshly saturated KHCO_3_ and introduced at a flow rate of 0.76 mL min^−1^ for 60 min. Linear sweep voltammetry from −0.3 V_RHE_ to −1.1 V_RHE_ (repeated twice) was first used to determine the onset potential for the CO_2_RR using a scan rate of 15 mV s^−1^ and to ensure that the applied potential was consistent between experiments, followed by chronoamperometry for up to 1 h at −0.9 V_RHE_ or −1.1 V_RHE_. The flow rate was maintained at 0.76 ml/min unless a bubble forms, at which point we increased the flow rate to 7.6 mL min^−1^ to push out the bubble. Due to the limited volume of 25 ml of our syringe, multiple syringe changes were required during the extended experiments. The intermittent bubble formation can be seen as jitters in the supplementary movies.

In situ imaging was always performed under conditions with electrolyte in the cell, as determined from the image contrast, and we stayed under an electron flux of 7 e^−^ Å^−2^ s^−1^ at all times to minimize electron beam-induced artifacts. Further control experiments to identify the influence of the electron beam are further discussed in Supplementary Note 4. The presence of liquid in the electrochemical cell was also confirmed using electron energy loss spectroscopy (a representative spectrum is provided in Supplementary Fig. 21). A rough estimate puts the liquid layer thickness at around 1 µm in our experiments. Also, at least two in situ experiments were performed at each size distribution for reproducibility.

The image segmentation for the in situ TEM movies was executed by using built-in functions and scripting in MATLAB. The analysis was performed in the following steps: (1) drift correction (2) bandpass filtering, (3) binarization, (4) particle detection, and (5) classification of the cubes and re-deposited particles. The band filter size and binary cutoff threshold were determined by visual inspection. For the NP size and NP number density plots in Fig. 4, each point is an average of 60 frames, and the error bars represent the standard deviation of 60 frames (segmentation error). The classification of the NP type was based on the particle size. A particle larger than a pre-determined size was categorized as a cube. Particles that were smaller than the threshold were categorized as re-deposited NPs. The size criteria were 200, 500, and 1000 pixels (57, 90, and 127 nm) for the Cu cubes of the average size of 80, 170, and 390 nm, respectively. The size criteria were determined by manual testing to find the size that resulted in the most correctly labeled particles. We also applied extra shape limiting criteria such that the eccentricity and the perimeter-to-area ratio, when necessary, to exclude the occasional mislabeling during segmentation.

### Ex situ transmission electron microscopy

The ex situ TEM imaging and STEM-EDX mapping were performed using a ThermoFisher 200 kV Talos F200X TEM. The EC-TEM chips were loaded on a Hummingbird Scientific Tomography holder with a customized tip for the chips. For the after-reaction samples, the electrochemical cells were rinsed in ultrapure water after they were disassembled from the holder and immediately transferred into the TEM.

### Scanning electron microscopy

The ex situ SEM imaging was performed using a ThermoFisher Apreo SEM with the in-lens secondary electron detector.

## Supplementary information

Supplementary Information

Supplementary Movie legends

Supplementary Movie 1

Supplementary Movie 2

Supplementary Movie 3

Supplementary Movie 4

Supplementary Movie 5

Supplementary Movie 6

Supplementary Movie 7

Supplementary Movie 8

Supplementary Movie 9

Supplementary Movie 10

Supplementary Movie 11

Supplementary Movie 12

Supplementary Movie 13

Supplementary Movie 14

## Data Availability

The authors declare that the data supporting the findings of this study are available within the paper and its Supplementary Information files. The raw data sets generated during the current study are available from the corresponding author on reasonable request.

## References

[1] Friend CM, Xu B (2017). Heterogeneous catalysis: a central science for a sustainable future. Acc. Chem. Res..

[2] De Luna P (2019). What would it take for renewably powered electrosynthesis to displace petrochemical processes?. Science.

[3] Handoko AD, Wei F, Jenndy, Yeo BS, Seh ZW (2018). Understanding heterogeneous electrocatalytic carbon dioxide reduction through operando techniques. Nat. Catal..

[4] Birdja YY (2019). Advances and challenges in understanding the electrocatalytic conversion of carbon dioxide to fuels. Nat. Energy.

[5] Nitopi S (2019). Progress and perspectives of electrochemical CO_2_ reduction on copper in aqueous electrolyte. Chem. Rev..

[6] Osowiecki WT (2019). Factors and dynamics of Cu nanocrystal reconstruction under CO_2_ reduction. ACS Appl. Energy Mater..

[7] Kim D (2017). Electrochemical activation of CO_2_ through atomic ordering transformations of AuCu nanoparticles. J. Am. Chem. Soc..

[8] Kim D, Kley CS, Li Y, Yang P (2017). Copper nanoparticle ensembles for selective electroreduction of CO_2_ to C2–C3 products. Proc. Natl Acad. Sci. USA.

[9] De Luna P (2018). Catalyst electro-redeposition controls morphology and oxidation state for selective carbon dioxide reduction. Nat. Catal..

[10] Grosse P (2018). Dynamic changes in the structure, chemical state and catalytic selectivity of Cu nanocubes during CO_2_ electroreduction: size and support effects. Angew. Chem. Int. Ed..

[11] Huang J (2018). Potential-induced nanoclustering of metallic catalysts during electrochemical CO_2_ reduction. Nat. Commun..

[12] Jung H (2019). Electrochemical fragmentation of Cu_2_O nanoparticles enhancing selective C-C coupling from CO_2_ reduction reaction. J. Am. Chem. Soc..

[13] Li Y (2020). Electrochemically scrambled nanocrystals are catalytically active for CO_2_-to-multicarbons. Proc. Natl Acad. Sci. USA.

[14] Arán-Ais RM, Scholten F, Kunze S, Rizo R, Roldan Cuenya B (2020). The role of in situ generated morphological motifs and Cu(i) species in C^2+^ product selectivity during CO_2_ pulsed electroreduction. Nat. Energy.

[15] Ross MB (2019). Designing materials for electrochemical carbon dioxide recycling. Nat. Catal..

[16] Popović S (2020). Stability and degradation mechanisms of copper-based catalysts for electrochemical CO_2_ reduction. Angew. Chem. Int. Ed..

[17] de Jonge N, Ross FM (2011). Electron microscopy of specimens in liquid. Nat. Nanotechnol..

[18] Ross FM (2015). Opportunities and challenges in liquid cell electron microscopy. Science.

[19] Taheri ML (2016). Current status and future directions for in situ transmission electron microscopy. Ultramicroscopy.

[20] Williamson MJ, Tromp RM, Vereecken PM, Hull R, Ross FM (2003). Dynamic microscopy of nanoscale cluster growth at the solid-liquid interface. Nat. Mater..

[21] Zhu GZ (2014). In situ liquid cell TEM study of morphological evolution and degradation of Pt-Fe nanocatalysts during potential cycling. J. Phys. Chem. C.

[22] Tan SF (2019). Intermediate structures of Pt-Ni nanoparticles during selective chemical and electrochemical etching. J. Phys. Chem. Lett..

[23] Beermann V (2019). Real-time imaging of activation and degradation of carbon supported octahedral Pt-Ni alloy fuel cell catalysts at the nanoscale using: In situ electrochemical liquid cell STEM. Energy Environ. Sci..

[24] Ortiz Peña N (2019). Morphological and structural evolution of Co_3_O_4_ nanoparticles revealed by in situ electrochemical transmission electron microscopy during electrocatalytic water oxidation. ACS Nano.

[25] Impagnatiello A (2020). Degradation mechanisms of supported Pt nanocatalysts in proton exchange membrane fuel cells: an operando study through liquid cell transmission electron microscopy. ACS Appl. Energy Mater..

[26] Arán-Ais RM (2020). Imaging electrochemically synthesized Cu_2_O cubes and their morphological evolution under conditions relevant to CO_2_ electroreduction. Nat. Commun..

[27] Vavra J, Shen T, Stoian D, Tileli V, Buonsanti R (2021). Real‐time monitoring reveals dissolution/redeposition mechanism in copper nanocatalysts during the initial stages of the CO_2_ reduction reaction. Angew. Chem. Int. Ed..

[28] Hodnik N, Dehm G, Mayrhofer KJJ (2016). Importance and challenges of electrochemical in situ liquid cell electron microscopy for energy conversion research. Acc. Chem. Res..

[29] Seh, Z. W. et al. Combining theory and experiment in electrocatalysis: insights into materials design. *Science* **355**, eaad4998 (2017). 10.1126/science.aad4998 28082532

[30] Trindell JA, Duan Z, Henkelman G, Crooks RM (2020). Well-defined nanoparticle electrocatalysts for the refinement of theory. Chem. Rev..

[31] Grosse P, Yoon A, Rettenmaier C, Chee SW, Cuenya BR (2020). Growth dynamics and processes governing the stability of electrodeposited size-controlled cubic Cu catalysts. J. Phys. Chem. C.

[32] Möller, T. et al. Electrocatalytic CO_2_ reduction on CuO_x_ nanocubes: tracking the evolution of chemical state, geometric structure, and catalytic selectivity using operando spectroscopy. *Angew. Chem. Int. Ed.*10.1002/anie.202007136 (2020). 10.1002/anie.202007136 PMC7590092 32627953

[33] Lin SC (2020). Operando time-resolved X-ray absorption spectroscopy reveals the chemical nature enabling highly selective CO_2_ reduction. Nat. Commun..

[34] de Jonge N, Houben L, Dunin-Borkowski RE, Ross FM (2019). Resolution and aberration correction in liquid cell transmission electron microscopy. Nat. Rev. Mater..

[35] Reske R, Mistry H, Behafarid F, Roldan Cuenya B, Strasser P (2014). Particle size effects in the catalytic electroreduction of CO_2_ on Cu nanoparticles. J. Am. Chem. Soc..

[36] Mistry H (2016). Tuning catalytic selectivity at the mesoscale via interparticle interactions. ACS Catal..

[37] Wang X, Varela AS, Bergmann A, Kühl S, Strasser P (2017). Catalyst particle density controls hydrocarbon product selectivity in CO_2_ electroreduction on CuO_x_. ChemSusChem.

[38] Liu, X. et al. Understanding trends in electrochemical carbon dioxide reduction rates. *Nat. Commun.* **8**, 15438 (2017). 10.1038/ncomms15438 PMC5458145 28530224

[39] Schneider NM (2014). Electron–water interactions and implications for liquid cell electron microscopy. J. Phys. Chem. C.

[40] Ambrožič B (2019). Controlling the radical-induced redox chemistry inside a liquid-cell TEM. Chem. Sci..

[41] Nicholls D (2020). Minimising damage in high resolution scanning transmission electron microscope images of nanoscale structures and processes. Nanoscale.

[42] Woehl TJ, Abellan P (2017). Defining the radiation chemistry during liquid cell electron microscopy to enable visualization of nanomaterial growth and degradation dynamics. J. Microsc..

[43] Speck, F. D. & Cherevko, S. Electrochemical copper dissolution: a benchmark for stable CO_2_ reduction on copper electrocatalysts. *Electrochem. Commun.* **115**, 106739 (2020).

[44] Gao D, Scholten F, Roldan Cuenya B (2017). Improved CO_2_ electroreduction performance on plasma-activated Cu catalysts via electrolyte design: halide effect. ACS Catal..

[45] Chang K (2020). Improving CO_2_ electrochemical reduction to CO using space confinement between gold or silver nanoparticles. J. Phys. Chem. Lett..

