# Author Correction: Dynamic transformations of cubic copper catalysts during CO_2_ electroreduction and its impact on catalytic selectivity

**DOI:** 10.1038/s41467-021-27500-4

**Published:** 2021-12-14

**Authors:** Philipp Grosse, Aram Yoon, Clara Rettenmaier, Antonia Herzog, See Wee Chee, Beatriz Roldan Cuenya

**Affiliations:** Department of Interface Science, Fritz-Haber Institute of the Max Planck Society, 14195 Berlin, Germany

**Keywords:** Electrocatalysis, Electrocatalysis, Nanoparticles

Correction to: *Nature Communicatio*ns 10.1038/s41467-021-26743-5, published online 18 November 2021.

The HTML version of this Article incorrectly omitted Supplementary Movie 1–14. Supplementary Movie 1–14 can be found as Supplementary Information associated with this Correction.

## Supplementary information

Supplementary Movie 1

Supplementary Movie 2

Supplementary Movie 3

Supplementary Movie 4

Supplementary Movie 5

Supplementary Movie 6

Supplementary Movie 7

Supplementary Movie 8

Supplementary Movie 9

Supplementary Movie 10

Supplementary Movie 11

Supplementary Movie 12

Supplementary Movie 13

Supplementary Movie 14

