# Supplementary material for: Dynamic transformation of cubic copper catalysts during CO2 electroreduction and its impact on catalytic selectivity
Source: Nat Commun. 2021 Nov 18;12:6736. doi: 10.1038/s41467-021-26743-5 (PMC8602378; doi:10.1038/s41467-021-26743-5)
Supplement: Supplementary file 1 — Supplementary Information [file 41467_2021_26743_MOESM1_ESM.pdf]

Supplementary Information for

Dynamic Transformation of Cubic Copper Catalysts during CO_2 Electroreduction and its Impact on Catalytic Selectivity

Philipp Grosse[+,1], Aram Yoon[+,1], Clara Rettenmaier[1], Antonia Herzog[1], See Wee Chee[1*] and Beatriz Roldan Cuenya[1*]

[1]Department of Interface Science, Fritz-Haber Institute of the Max Planck Society, Berlin 14195

e-mail: swchee@fhi-berlin.mpg.de; roldan@fhi-berlin.mpg.de

[+] These authors contributed equally to the work.

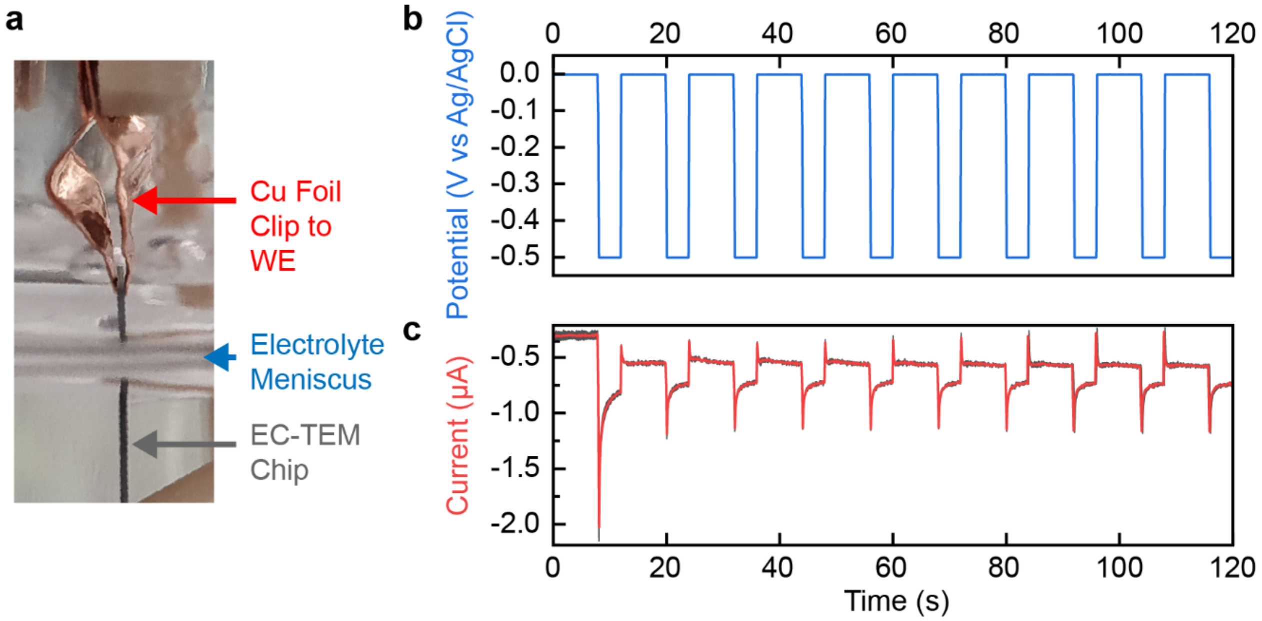

Supplementary Figure 1. ***Ex situ*** **synthesis of Cu_2O cubes on EC-TEM chip.** (a) Photograph of our setup for the electrodeposition of cubes on the EC-TEM chips in a standard H-type cell. Contact to the carbon working electrode (WE) of the chips is made using copper foil clips. (b) Alternating potential profile used for the electrochemical deposition. (c) Corresponding current traces obtained from four different depositions using the same synthesis conditions.

Supplementary Table 1: Average sizes of Cu_2O cubes on the EC-TEM chip and on glassy carbon (averaged over 3-10 sample) and three sets of sample sizes & loading to demonstrate reproducibility with our electro-deposition method. The scale bars are 500 nm.

**On EC-TEM chip**					
**Name**	**Size in nm**	**StDev in nm**	**Size & Loading sample #1**	**Size & Loading sample #2**	**Size & Loading sample #3**
**"80 nm"**	83±11	12±3	80±18 nm @ 25% 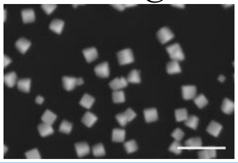	83±10 nm @ 39% 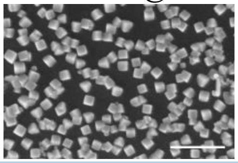	84±10 nm @ 39% 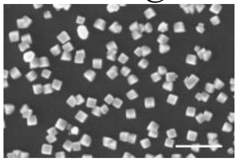
**"170 nm"**	158±25	47±17	185±34 nm @ 11% 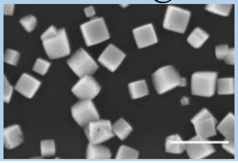	193±20 nm @ 9% 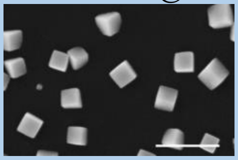	164±71 nm @ 11% 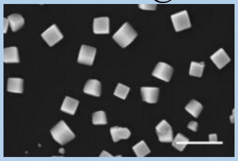
**"390 nm"**	343±39	11±6	312±13 nm @ 13% 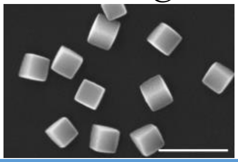	351±17 nm @ 8% 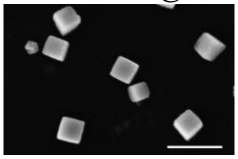	306±11 nm @ 10% 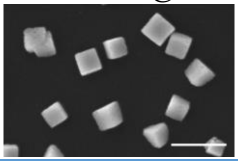
**On glassy carbon**					
**Name**	**Size in nm**	**StDev in nm**	**Size & Loading sample #1**	**Size & Loading sample #2**	**Size & Loading sample #3**
**"80 nm"**	87±9	13±1	89±6 nm @ 31% 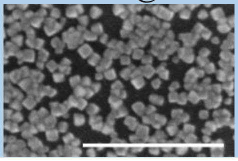	88±7 nm @ 27% 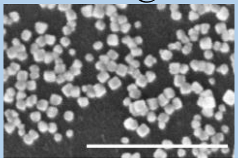	80±7 nm @ 32% 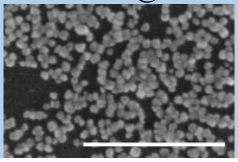
**"170 nm"**	177±7	29±1	187±24 nm @ 21% 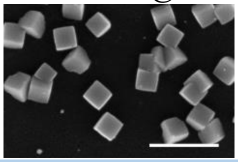	169±18 nm @ 17% 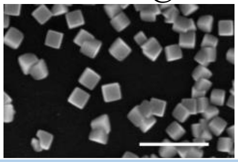	175±26 nm @ 19% 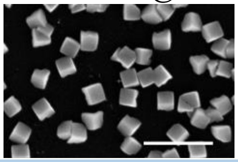
**"390 nm"**	359±22	41±2	379±44 nm @ 9% 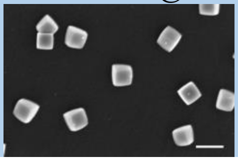	371±39 nm @ 7% 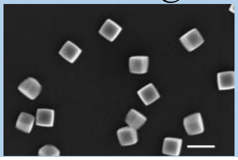	328±40 nm @ 1% 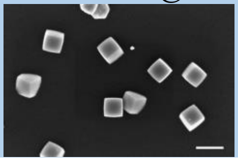

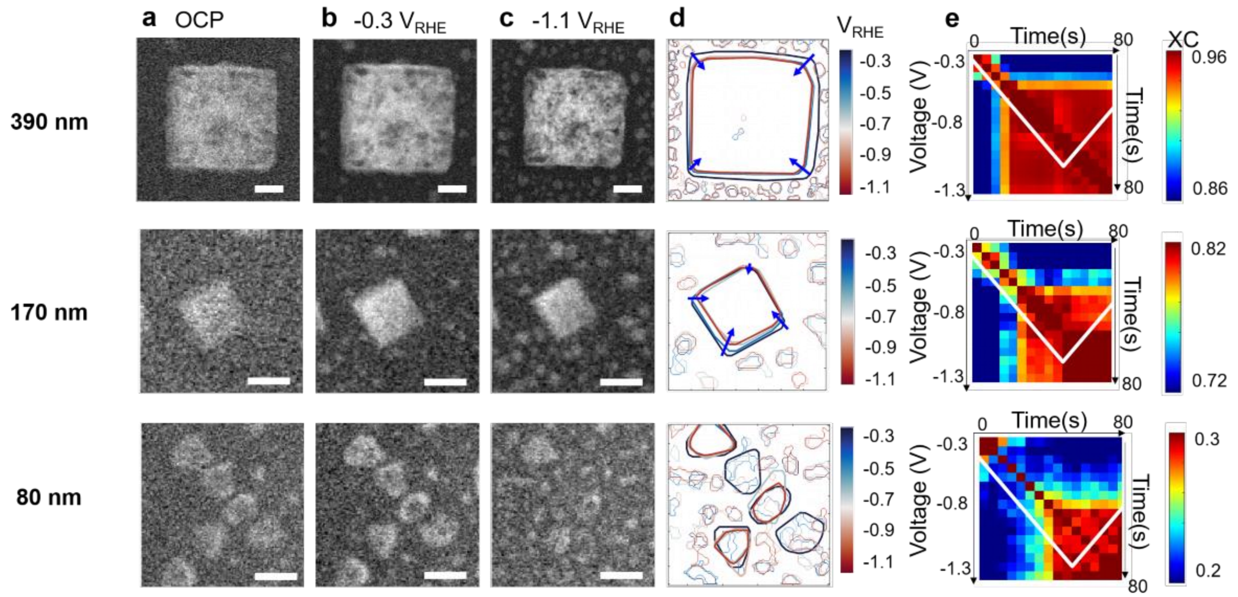

Supplementary Figure 2. Cube size changes and particle re-deposition during the potential sweep from open circuit potential. TEM images of Cu cubes (a) at OCP, (b) at -0.3 V_{RHE} and (c) at -1.1 V_{RHE} showing the fragmentation of the cubes and the formation of re-deposited particles. (d) Line profiles showing the outlines of cubes and re-deposited particles at different applied potentials. The arrows highlight the shrinking of the cubes. (e) Cross correlation coefficient (XC) maps comparing each image frame and its subsequent frame. The more similar each pair of images are, the higher the XC number. The white line indicates the applied potential over time. The maps show that the changes (areas in blue) occur mostly at the beginning of the potential sweep (-0.3 V_{RHE}) and up to -0.7 V_{RHE}, where re-deposited particles form and the cubes shrink respectively. The morphology is relatively stable over the rest of the voltage sweep (areas in red). The images in column (a) are raw frames that are not averaged whereas the other two columns (b and c) are obtained by averaging 5 adjacent frames in the image sequence.

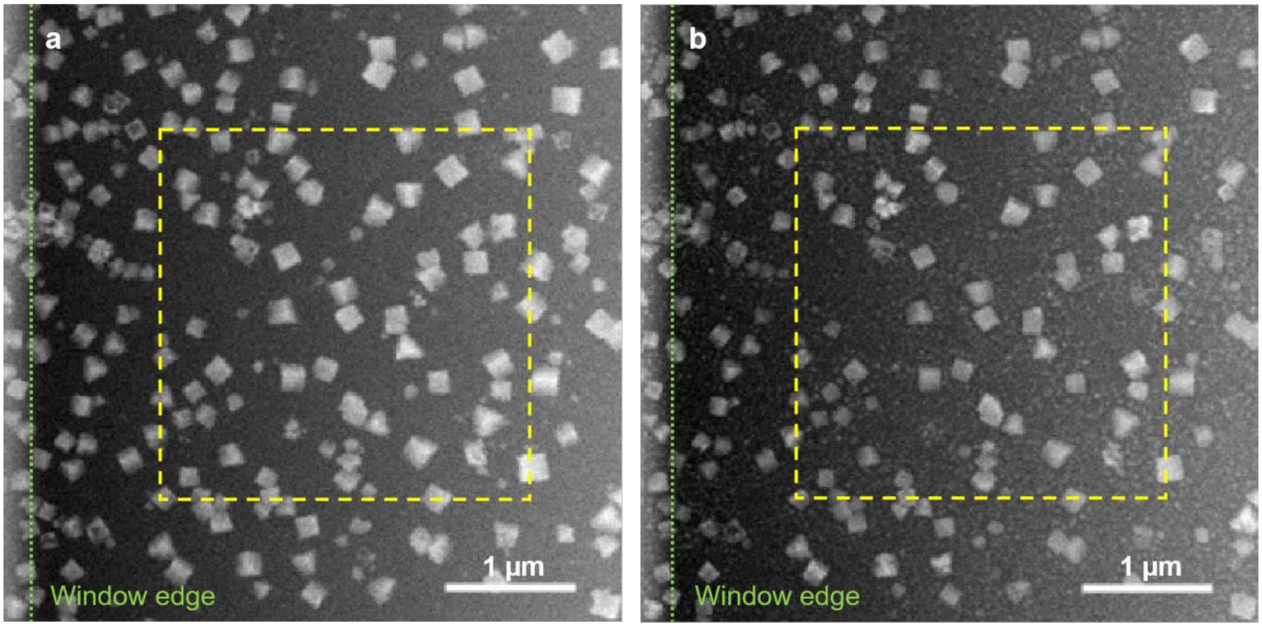

Supplementary Figure 3. Comparison of catalysts on the working electrode before and after a linear potential sweep to -1.1 V_{RHE}. Slow-scan STEM images taken from a larger field (a) before and (b) after sweeping the potential. The yellow dashed boxes indicate the area imaged during the in situ experiment shown in Figure 1 of the main text. Note that the better contrast and resolution of the area near the window edge (thinner liquid layer) indicate the absence of small re-deposited particles before the potential is applied. The observation of re-deposited particles extending beyond the in situ imaged area after the experiment also confirms that the re-deposition of particles is caused by the applied potential, and not due to beam-induced effects.

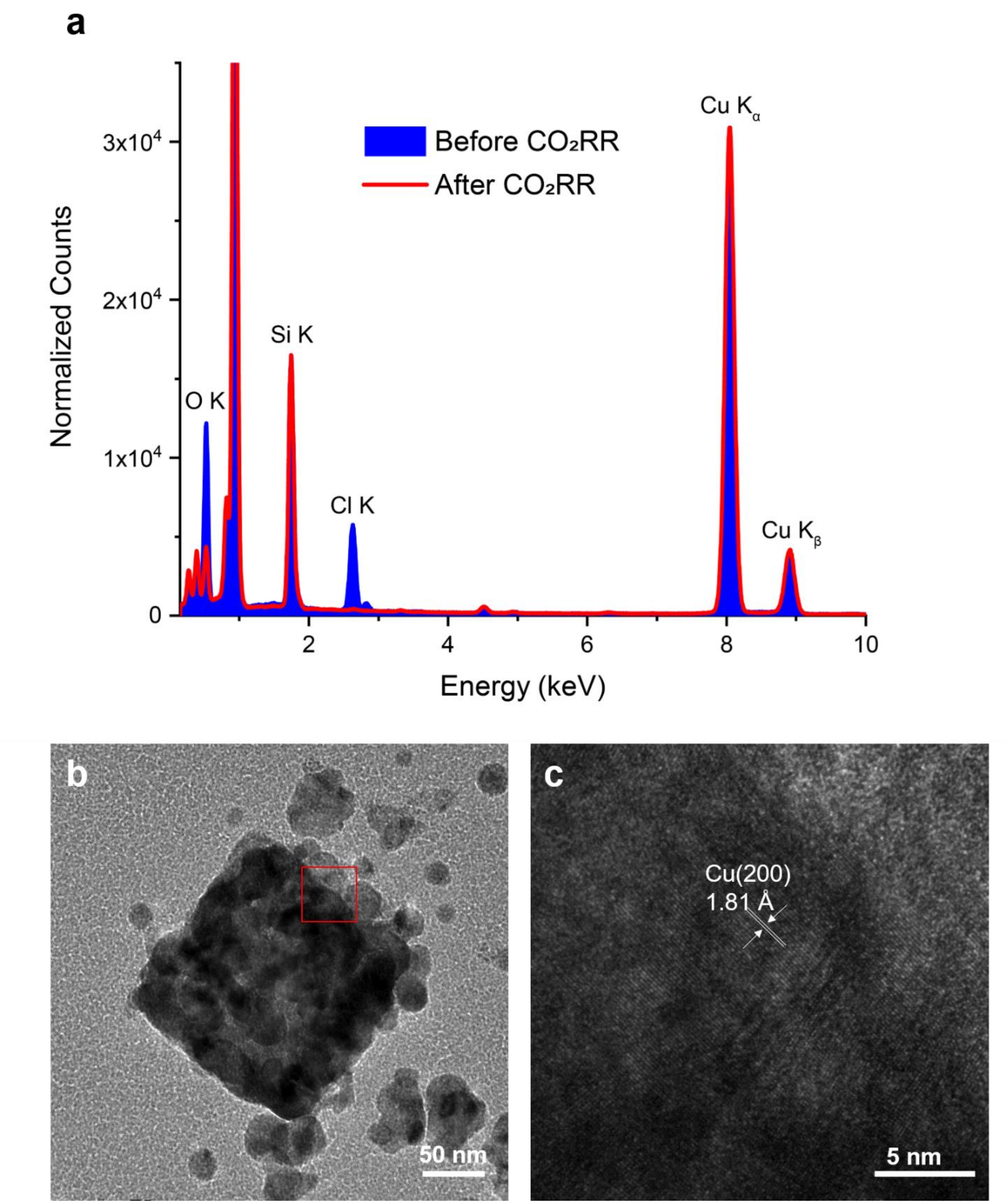

Supplementary Figure 4. Copper reduction after CO_2RR (a) Comparison of the EDX spectra obtained during the acquisition of Figure 1(c). The results indicate a decrease in both Cl and O after reaction. The spectra are normalized to the Si K peak of the silicon nitride membrane. (b) Cu cubes after CO_2RR. (c) The (100) facet of the magnified area within the Cu NP marked by a red square in (b). The lattice parameter 1.81 Å corresponds to Cu(200).

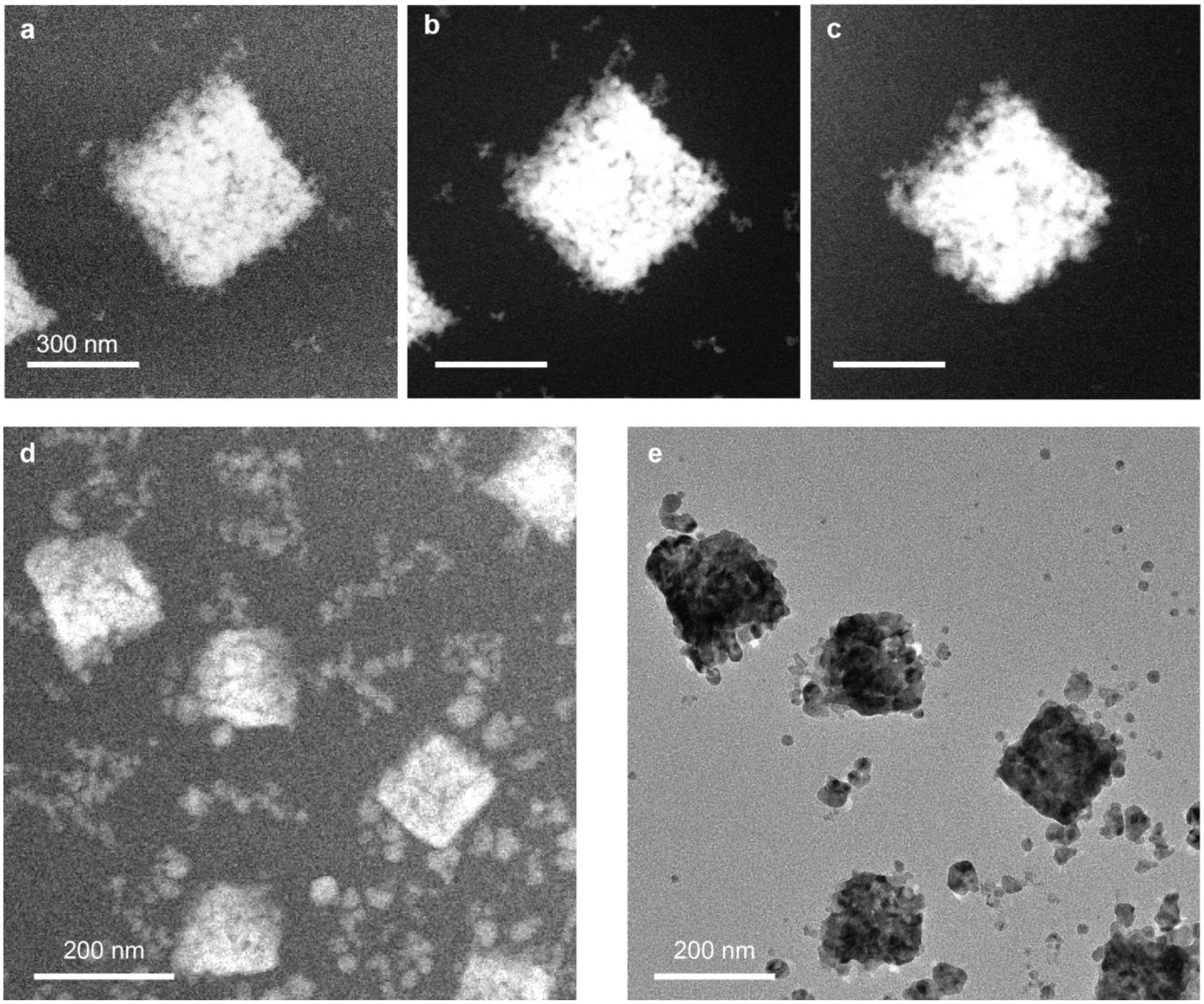

Supplementary Figure 5. Loss of re-deposited particles during and after *in situ* experiments. STEM images showing an experiment where (a) re-deposited NPs were initially seen but were sweep away after (b) a bubble formed and (c) was subsequently pushed out with a higher injection rate on the syringe pump. Images comparing of (d) the end of an *in situ* experiment where the re-deposited NPs could be seen at but were gone after (e) the liquid cell was dis-assembled and the chip was rinsed for *ex situ* measurements. Notice that the cube in the upper right corner of (d) also detached.

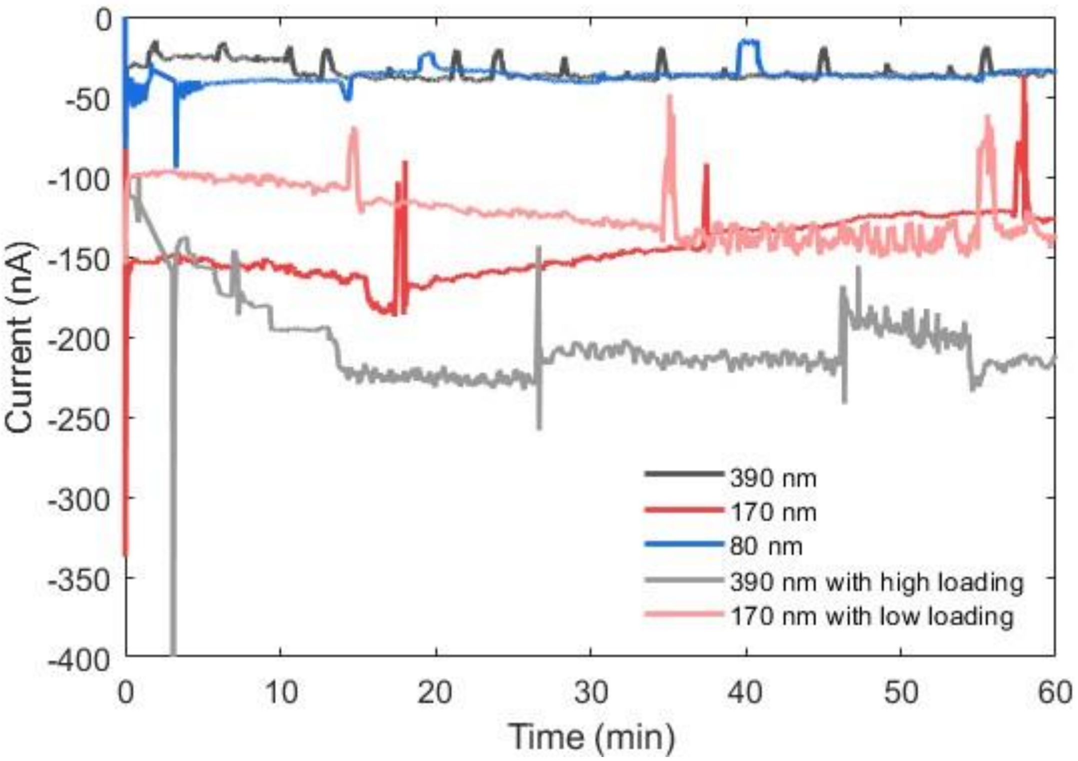

Supplementary Figure 6. Representative plots of currents measured during the EC-TEM chronoamperometric experiments. In general, currents are consistent with the catalyst density observed during the experiments, but there were some differences between similar experiments, which we attribute to variation in flow conditions between different liquid cells as reported previously[1]. The spikes in the currents are due to the stalling of the syringe pump caused by an empty syringe.

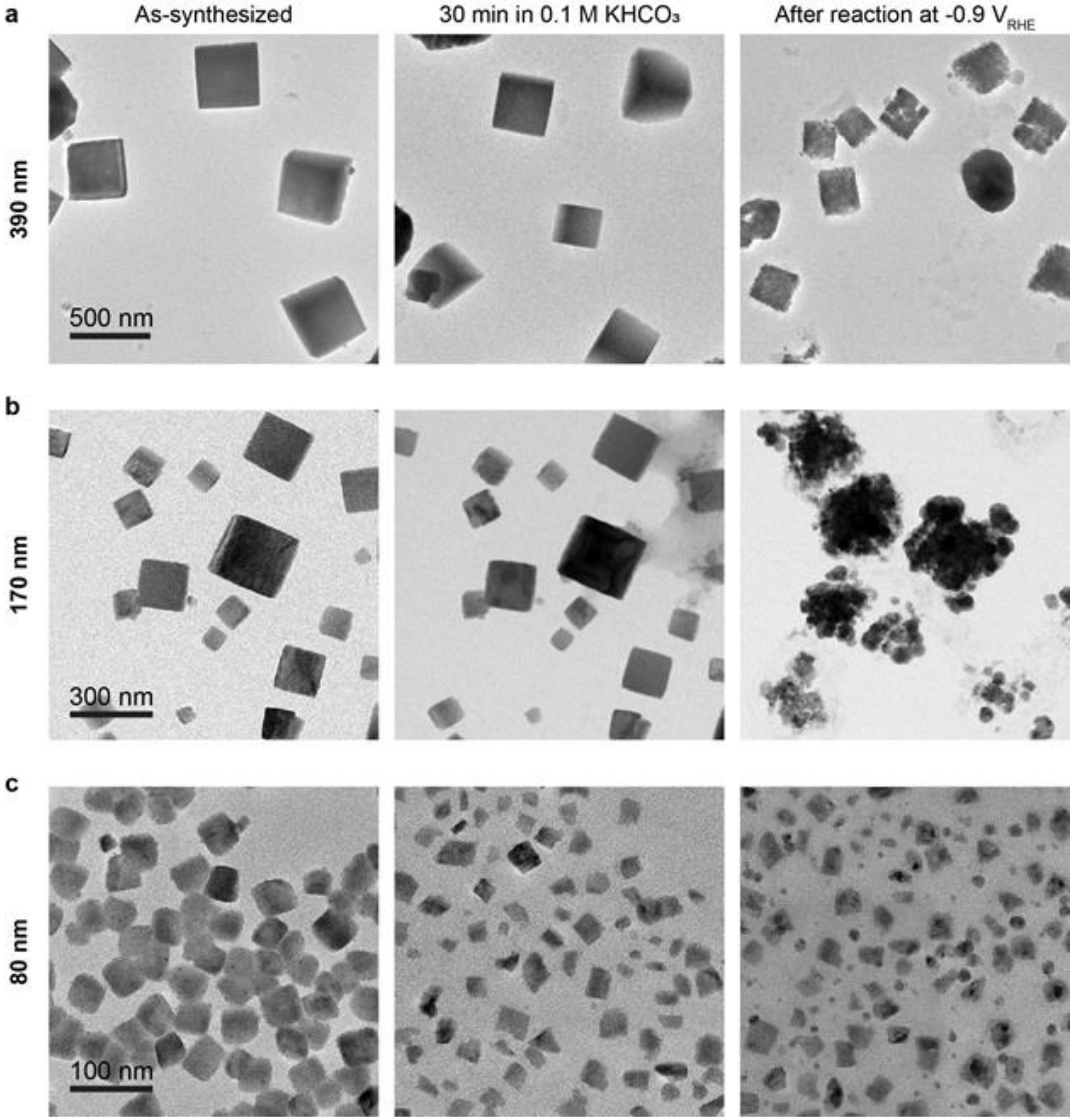

Supplementary Figure 7. *Ex situ* TEM images of areas comparing Cu_2O cubes synthesized on the EC-TEM chip but subject to CO_2RR in the benchtop setup. Image sequences comparing (a) 390 nm, (b) 170 nm and (c) 80 nm cubes. To mimic the *in situ* experiments, the chips were immersed in 0.1 M $KHCO_3$ (not connected) for 30 minutes followed by 1 hour reaction in CO_2-saturated 0.1 M $KHCO_3$ at -0.9 V_{RHE}. A comparison of the images acquired after immersion in 0.1 M $KHCO_3$ of the 80 nm cubes also suggests that the introduction of $KHCO_3$ alter the morphology and surface density of these smaller cubes. Our previous work indicates that more subtle changes occur in the surface morphology of the larger cubes[2], but these changes are difficult to see from TEM images.

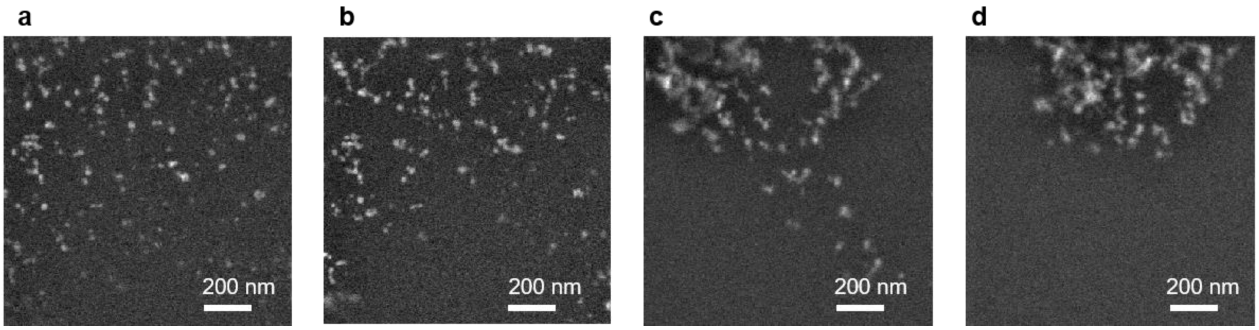

Supplementary Figure 8. Additional EC-TEM image sequence of 80 nm cubes from an area where there were mostly fragments and redeposited NPs acquired during CO_2RR at (a) 0 min, (b) 5 min, (c) 25 min, and (d) 45 min in CO_2-saturated 0.1 M $KHCO_3$ at -0.9 V_{RHE}. Image sequence was acquired with an electron flux of 3.5 e^- $Å^{-2}$ s^{-1}.

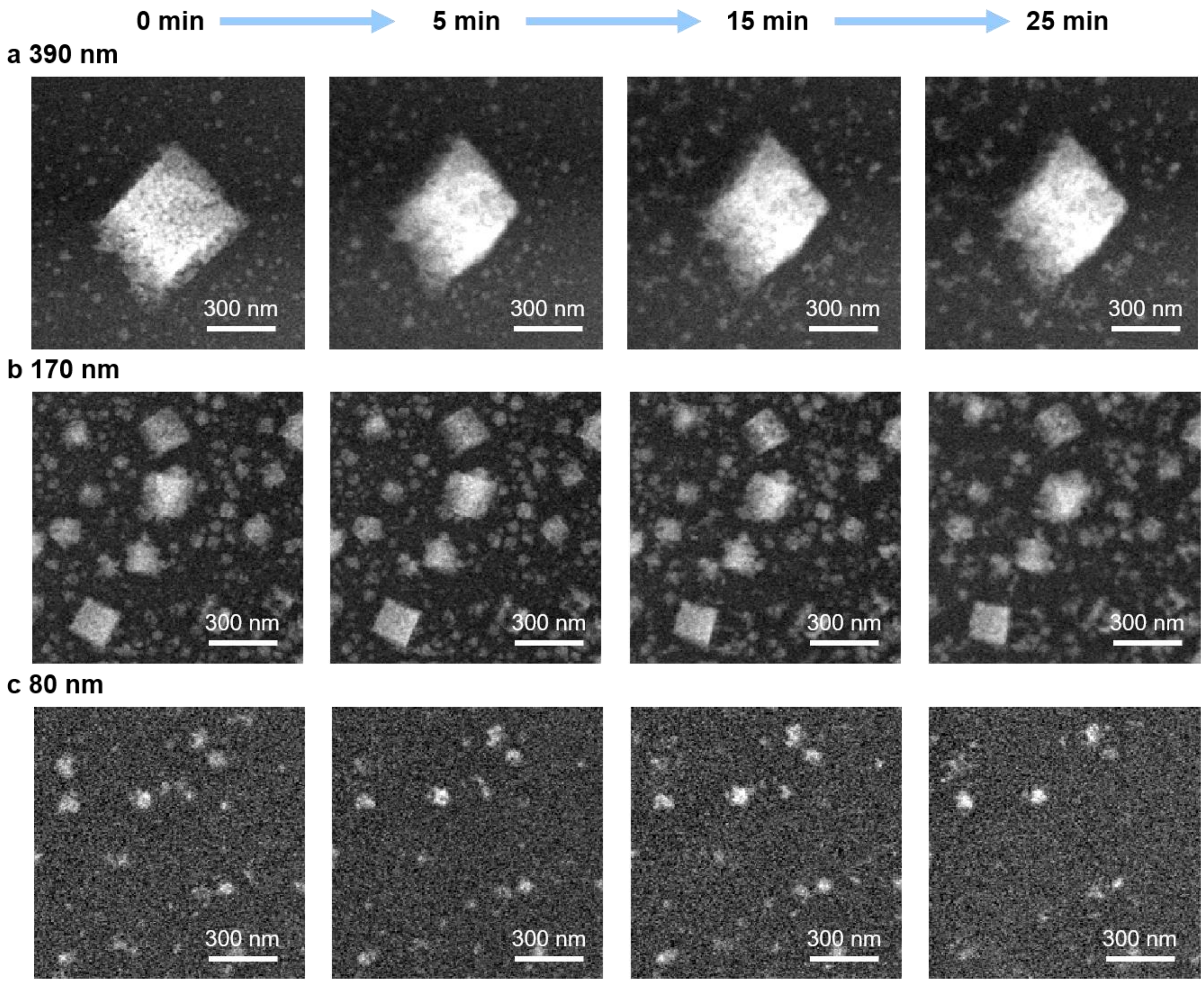

Supplementary Figure 9. Additional EC-TEM experiments with initial cube size and loading similar to the samples shown in Figure 2 but acquired at a lower electron flux. Images sequences of (a) 390 nm cubes (Supplementary Movie 8), (b) 170 nm cubes (Supplementary Movie 9), and (c) 80 nm cubes (Supplementary Movie 10) acquired in CO_2-saturated 0.1 M $KHCO_3$ at -0.9 V_{RHE} with an electron flux of 1.7 e^{-} $Å^{-2}$ s^{-1}.

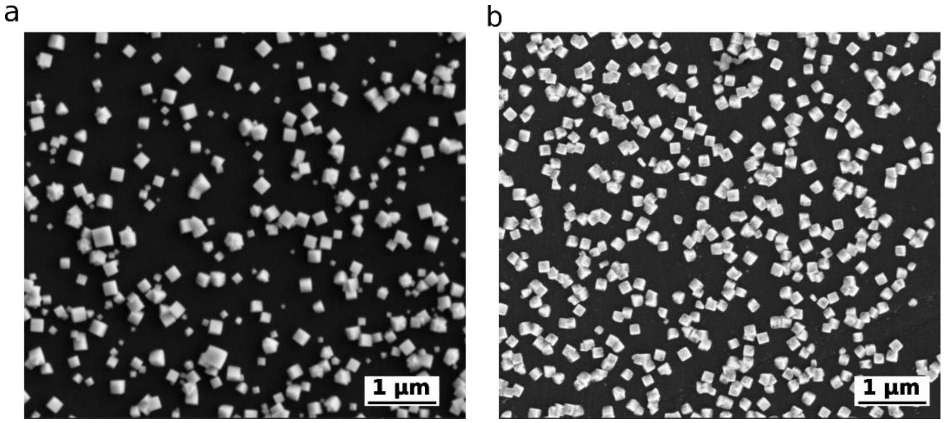

Supplementary Figure 10. Comparison of 170 nm Cu_2O cubes deposited on the carbon-coated EC-TEM chip electrode and a glassy carbon electrode for benchtop experiments. Generally, the Cu cubes on the chip (a) have a broader size distribution and lower loading that the cubes on the glassy carbon plate (b).

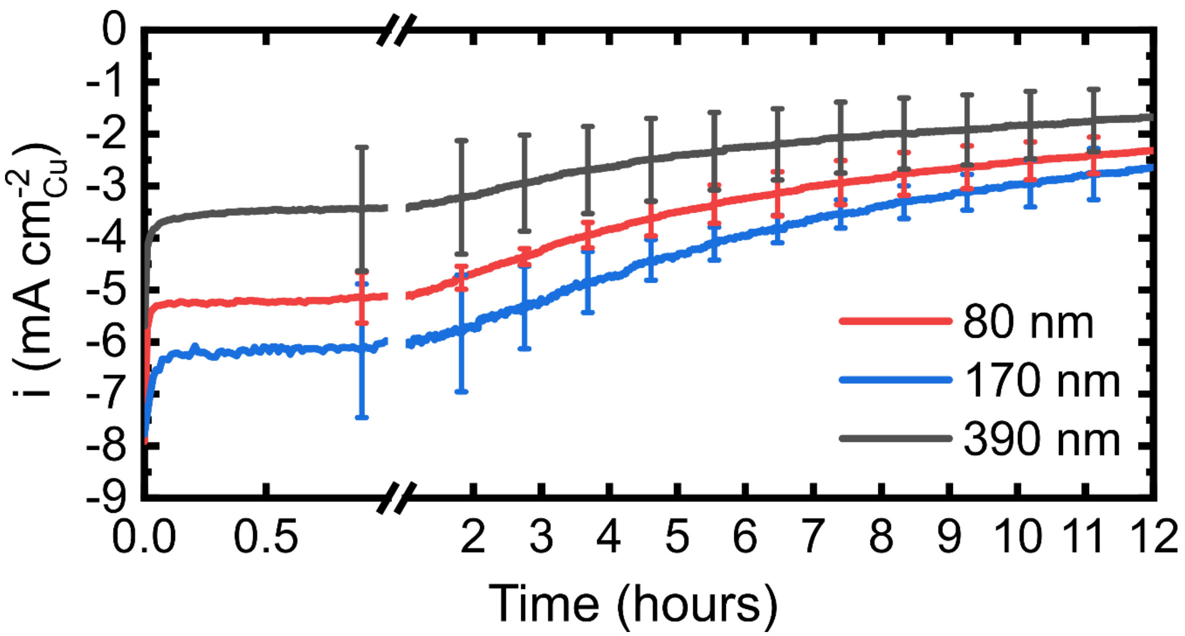

Supplementary Figure 11. Chronoamperometric traces obtained during the benchtop time-resolved product analysis experiments. The plot shows the chronoamperometric traces for 80 nm, 170 nm, and 390 nm Cu cubes on glassy carbon plates measured for 12 h at -1.1 V_{RHE}. Each trace is the average of three independent measurements. The error bars give the standard deviation of three measurements.

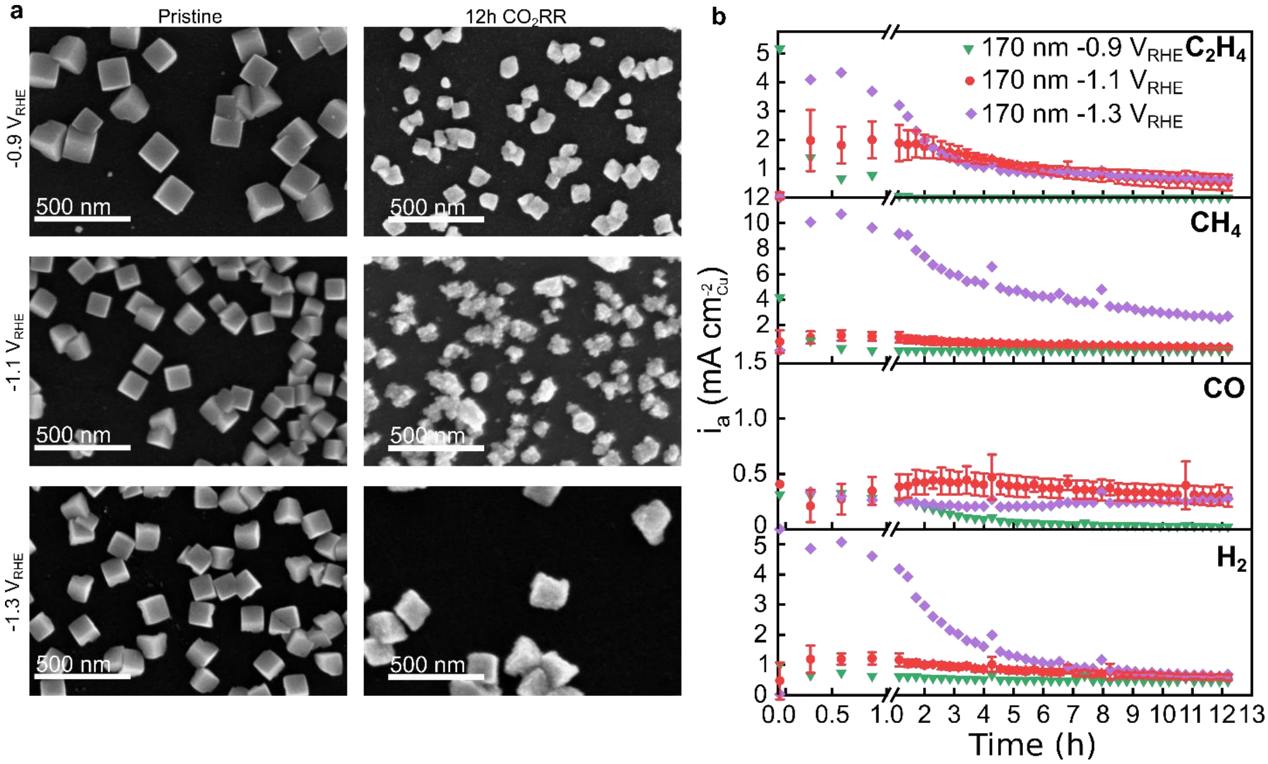

Supplementary Figure 12. Comparison of the time-dependent evolution of the morphology of 170 nm cubes and gaseous products of CO_2RR at different reduction potentials. (a) *Ex* situ SEM images of Cu_2O cubes deposited on glassy carbon plates before and after 12 hours of the reaction. Comparison of Cu cubes deposited on a glassy carbon plate before (pristine) and after CO_2RR at -0.9 V_{RHE}, -1.1 V_{RHE} and -1.3 V_{RHE}. It should be mentioned that the after-reaction images at -1.3 V_{RHE} also indicate that there was detachment and aggregation of these larger cubes, similar to what we observed in the 80 nm cubes at -0.9 V_{RHE}, as described in the main text. (b) Partial current density of 170 nm Cu cubes measured at -0.9 V, -1.1 V, and -1.3 V_{RHE} for CO, H_2, CH_4, and C_2H_4. The biggest changes were observed within the first hour of reaction, with secondary long term stability changes. The error bars give the standard deviation of three measurements.

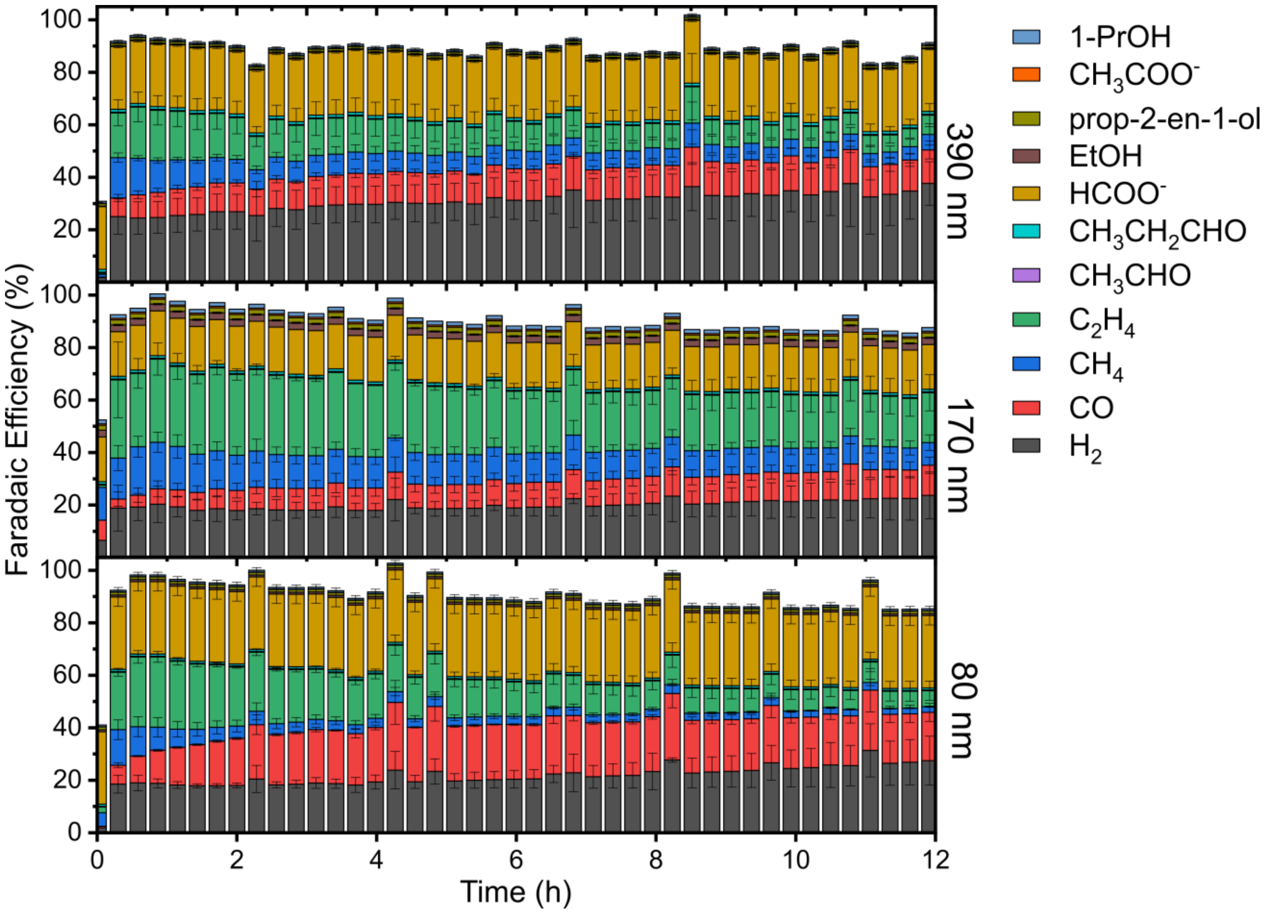

Supplementary Figure 13. Full product analysis over 12 hours of CO_2RR with Cu_2O cubes on glassy carbon plates. Gaseous products were measured via online gas chromatography during the reaction and liquid products were measured with high performance liquid chromatography (HPLC) after the reaction. The error bars give the standard deviation of three measurements.

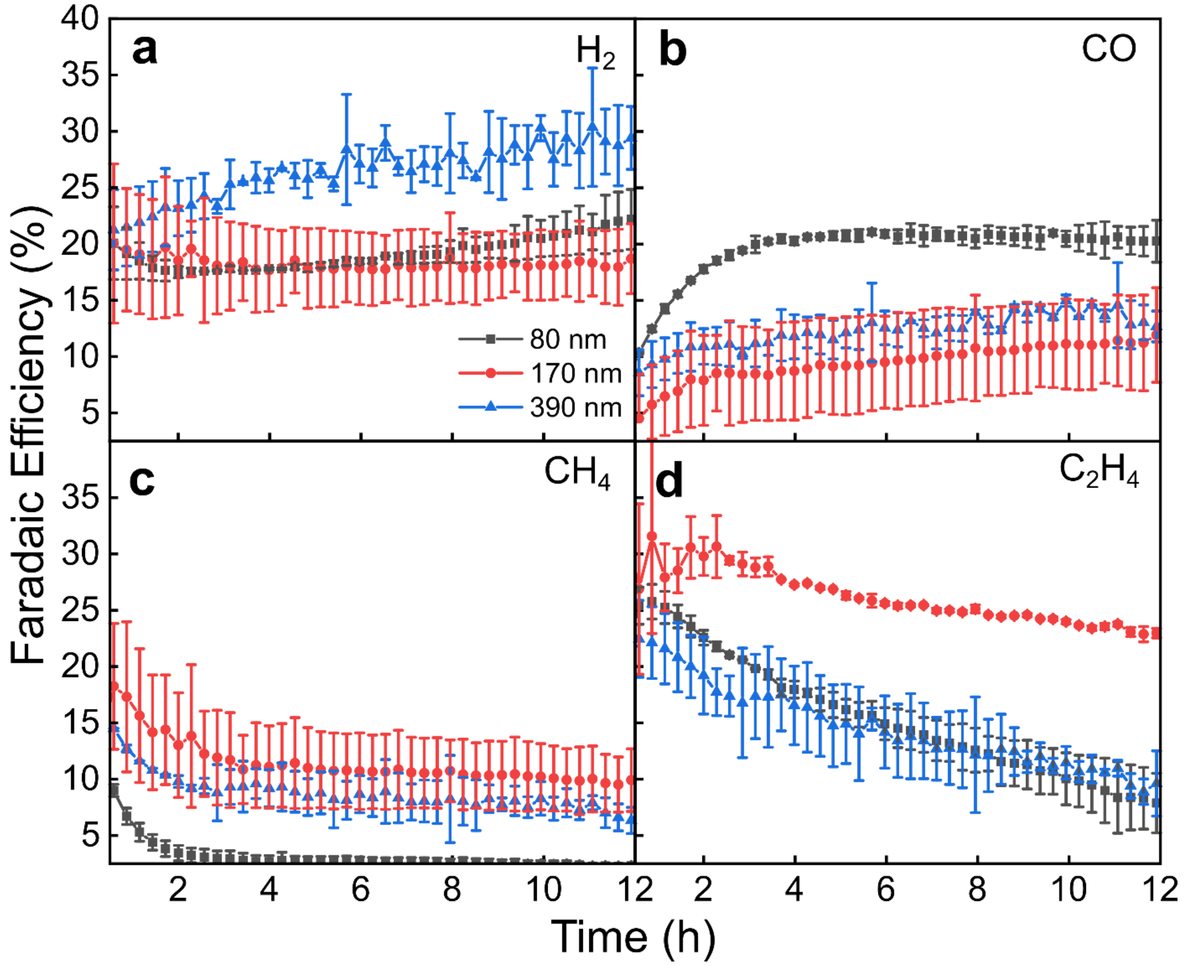

Supplementary Figure 14. Comparison of the time-dependent CO_2RR Faradaic Efficiency evolution for gaseous products. Faradaic efficiencies of three cube sizes (80 nm, 170 nm, 390 nm) for (a) hydrogen, (b) carbon monoxide, (c) methane, and (d) ethylene. The error bars give the standard deviation of three measurements.

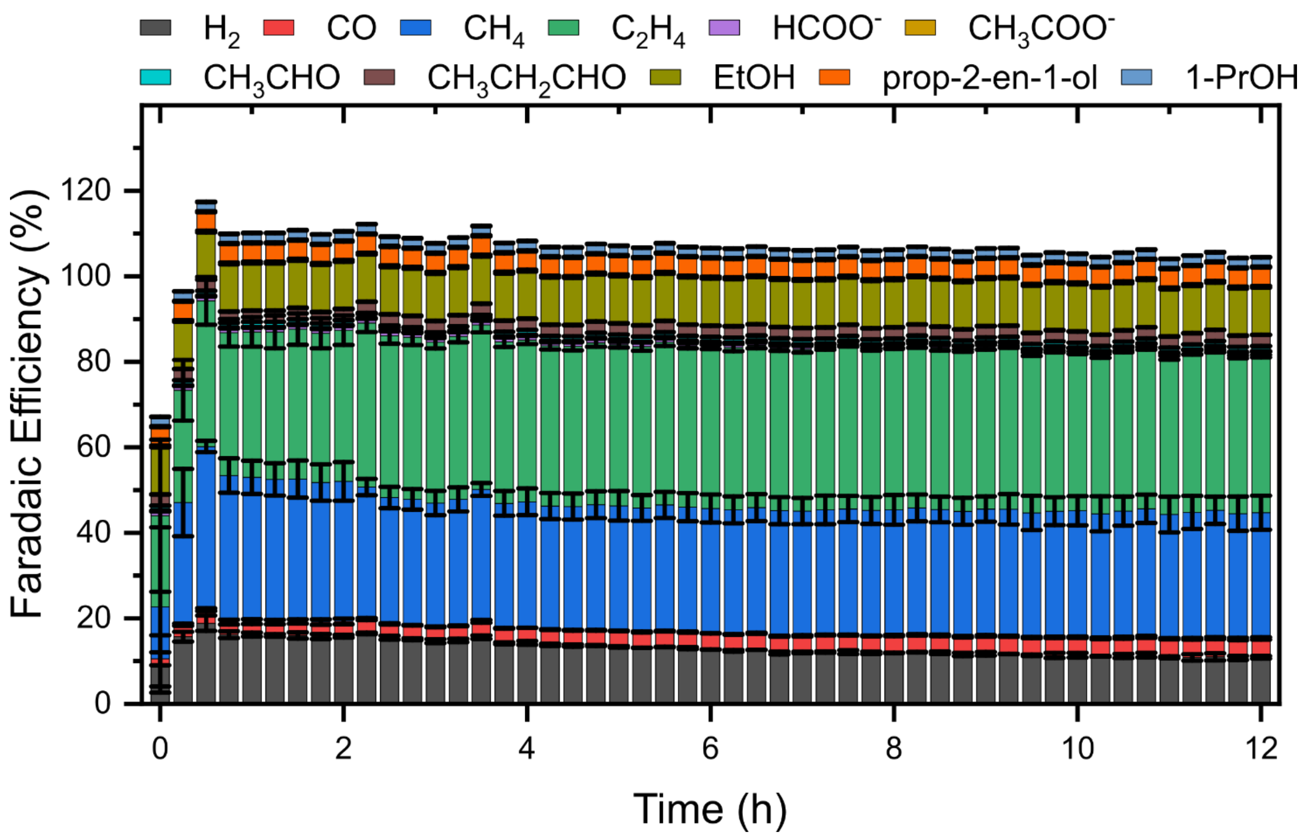

Supplementary Figure 15: Full product analysis for ~390 nm Cu cubes electrodeposited on glassy carbon at 3x higher loading than the analogous sample shown in the main text measured for 12h at -1.1 V_{RHE} in 0.1 M $KHCO_3$. We observed superior stability as well as suppressed CO and H_2 formation in favor of CH_4 and C_2H_4. The error bars give the standard deviation of three measurements.

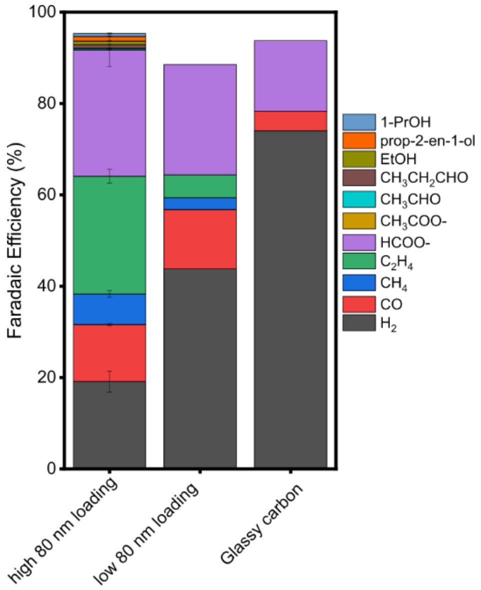

Supplementary Figure 16: Comparison of the Faradaic efficiencies obtained after 1 h of CO_2RR at -1.1 V_{RHE} of a sample containing a lower loading of 80 nm Cu_2O cubes with the 80 nm cubes in Figure 3 and a glassy carbon plate. The sample labelled as "lower loading" has ~50% less cubes than the samples described in the main text. The signals of the liquid products for the lower loading samples were below the detection limit. The hydrocarbon production significantly decreased with the reduced loading. The error bars give the standard deviation of three measurements.

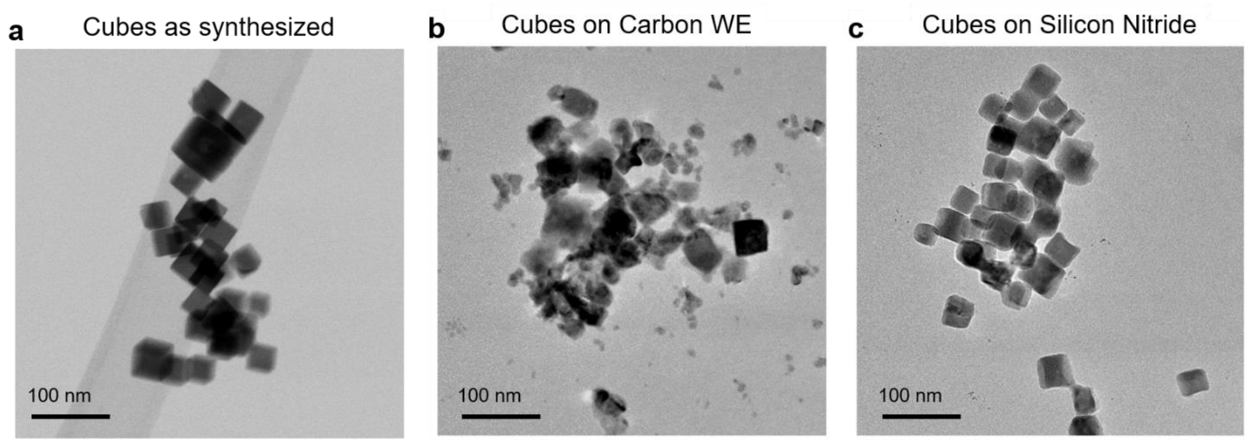

Supplementary Figure 17. *Ex situ* TEM images of 50 nm Cu_2O cubes synthesized with colloidal chemistry. (a) Image of the as-synthesized cubes. Images of cubes after 1 hour of CO_2RR that are found in two areas of the silicon nitride window: (b) the carbon WE of the EC-TEM chip and (c) the bare silicon nitride membrane. Images on the carbon WE show significant restructuring and formation of small NPs whereas the cubes on the silicon nitride show only small changes in their shape (presumably un-reacted due to the absence of electrical contact).

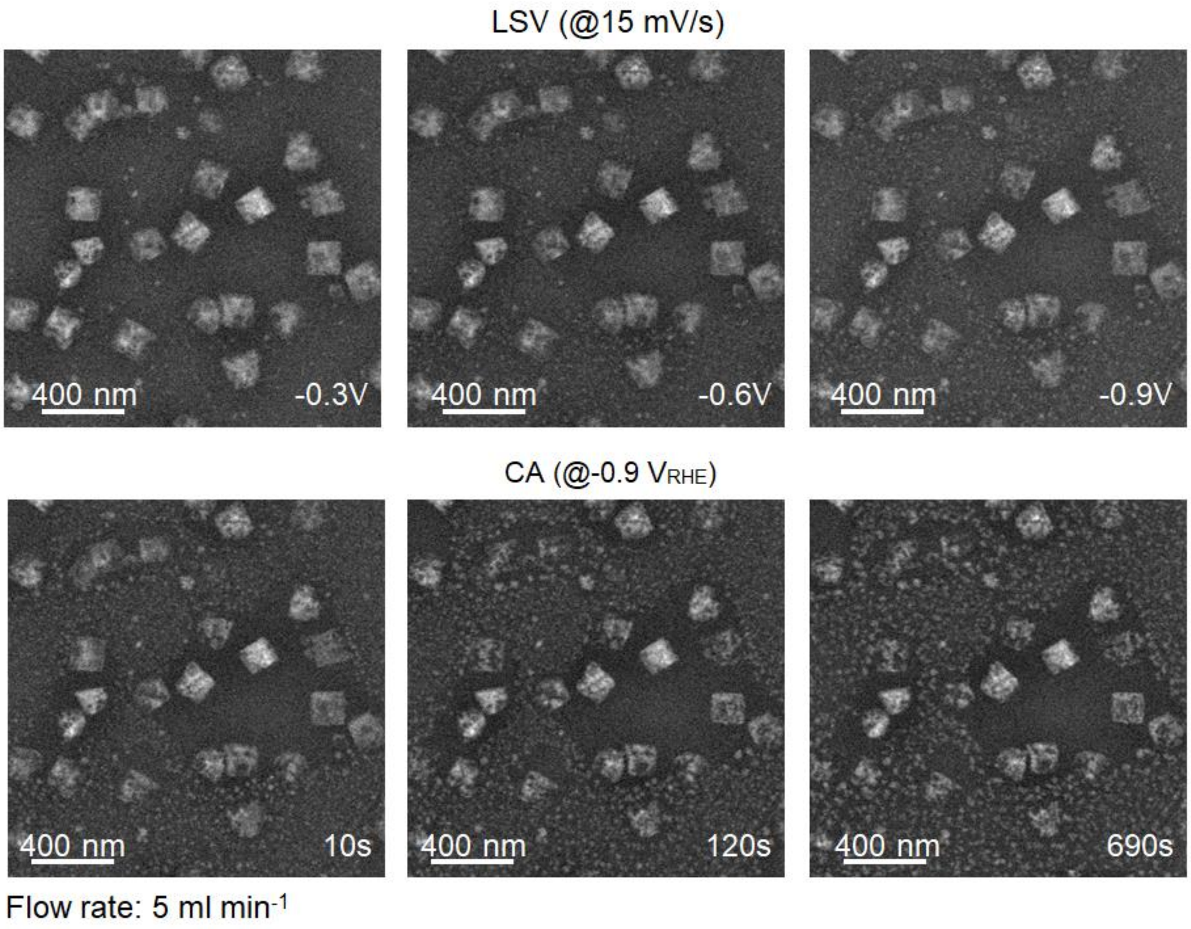

Supplementary Figure 18. Dynamics of the 170 nm of Cu_2O cubes obtained at high electrolyte flow velocities. Image sequences acquired under higher electrolyte velocity (5 ml/min). The scan rate during the linear sweep was 15 mV/s and the applied constant potential during chronoamperometry was -0.9 V_{RHE}. The electron flux used was 3.5 e^{-} $Å^{-2}$ s^{-1}.

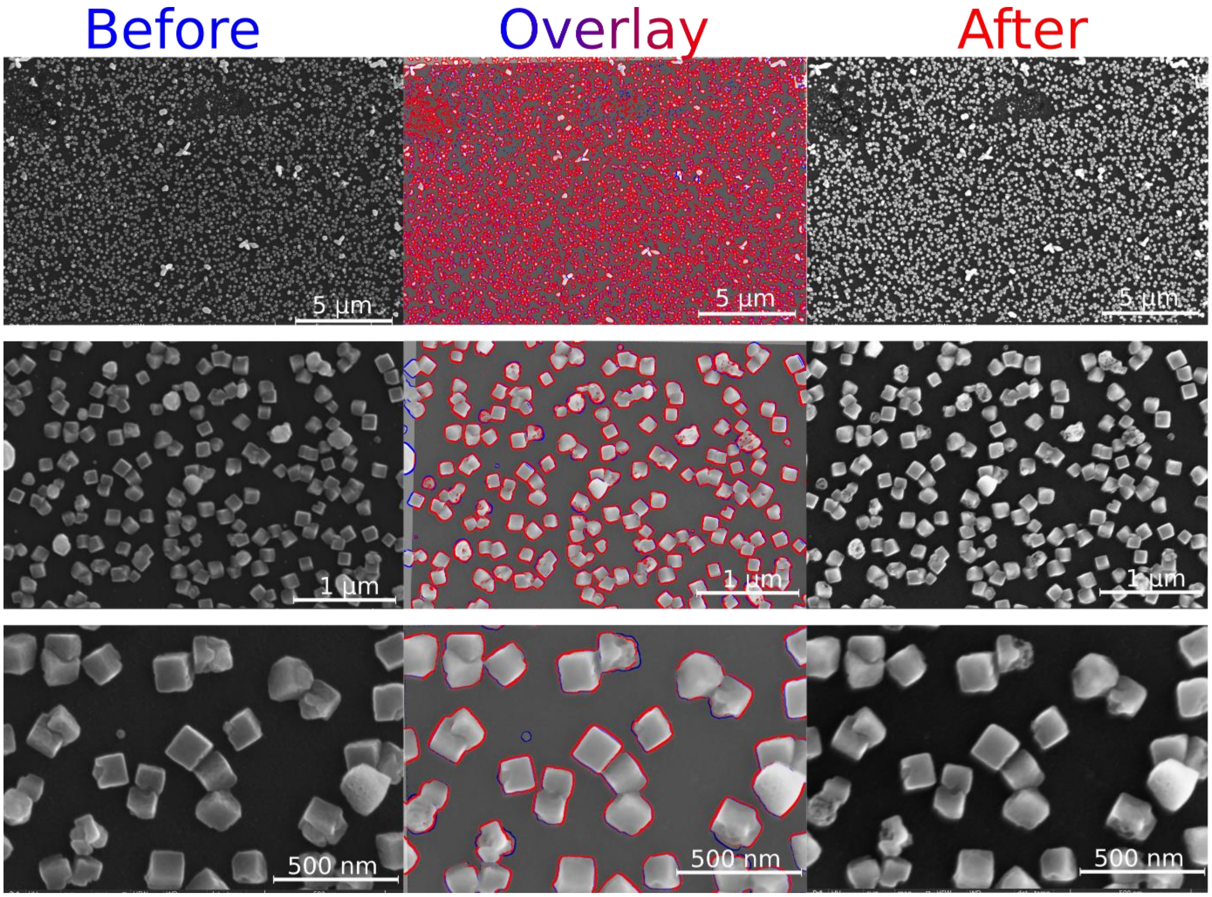

Supplementary Figure 19: Identical location SEM images comparing the same 170 nm cubes before and after 12 hours of $KHCO_3$ exposure. The middle column presents an overlay of the SEM images acquired before (blue), and after (red) $KHCO_3$ exposure There were no significant changes observed in the morphology and coverage due to the extended $KHCO_3$ exposure in the absence of an applied potential, suggesting that the dissolution is only limited to the cube surface and the initial electrolyte exposure.

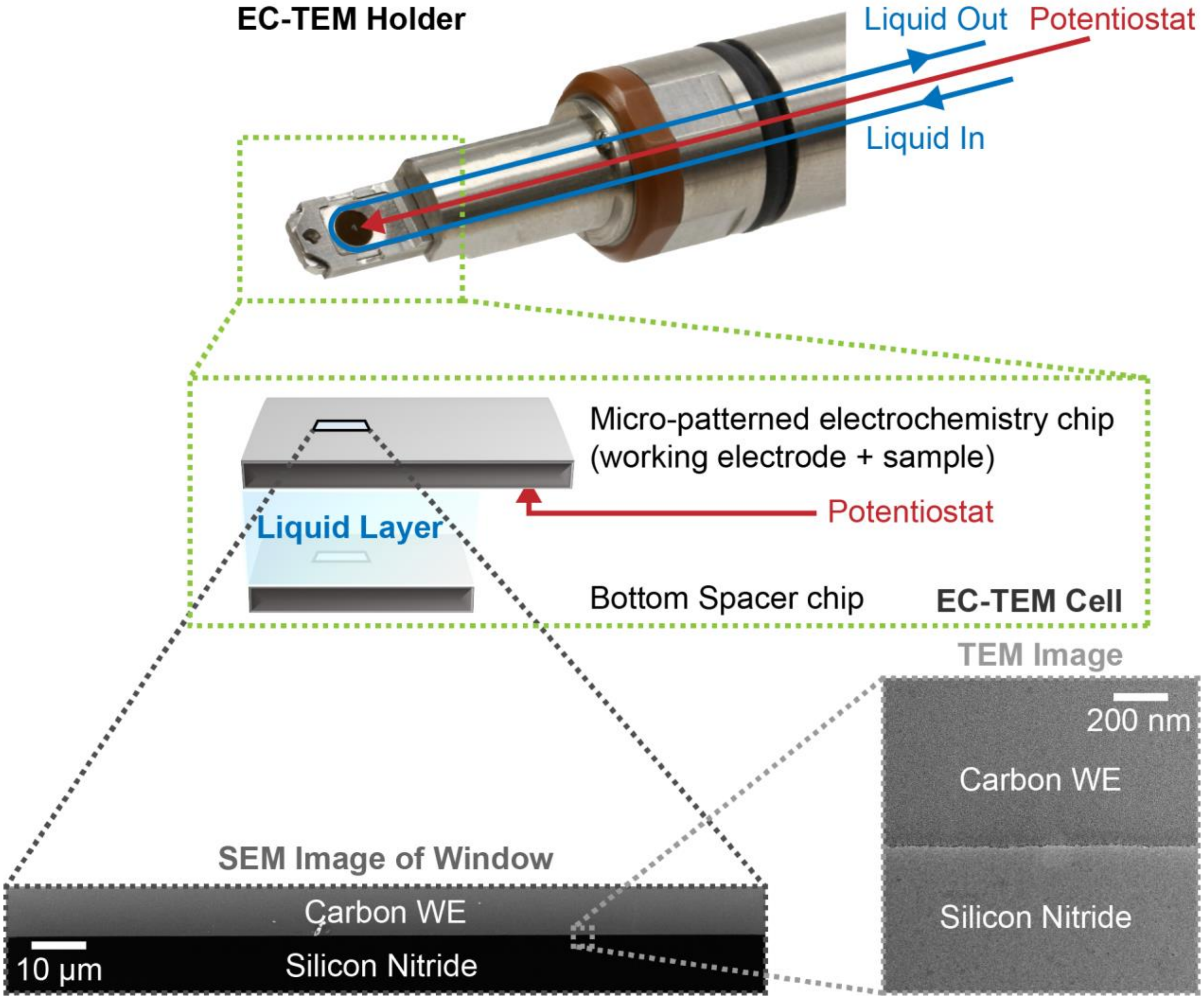

Supplementary Figure 20. Schematic of the experimental geometry. The *in situ* imaging is achieved by encapsulating the electrolyte in between two chips with electron-transparent silicon nitride membrane windows. The electrolyte is flowed into the cell using a syringe pump, whereas the electrochemical biasing is achieved through a micropatterned carbon film that is connected to the working electrode of a potentiostat. Image of the TEM holder is courtesy of Hummingbird Scientific.

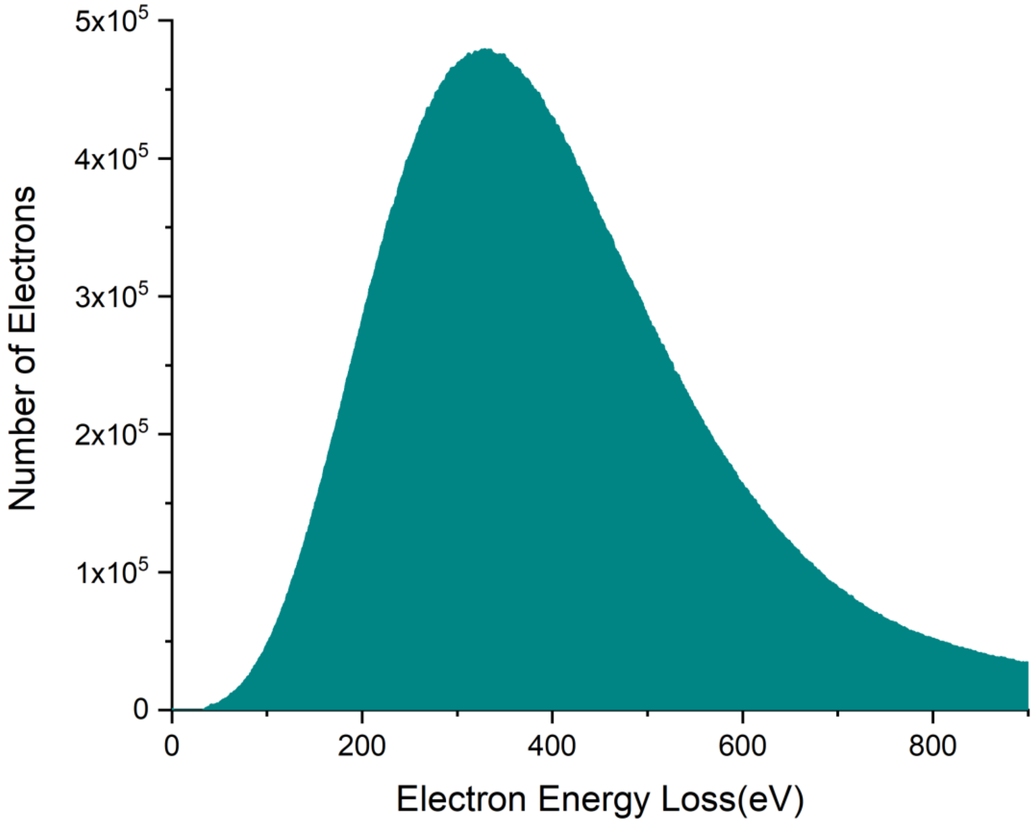

Supplementary Figure 21. A representative EELS spectrum acquired during our *in situ* TEM experiments. The profile of the spectrum indicates significant scattering of the primary electrons as they propagate through the electrochemical cell. Note the absence of the zero-loss peak, which indicates that the liquid thickness exceeds at least a few inelastic mean free paths of the 300 kV electrons. The features of the spectrum indicate that we have a liquid layer thickness of more than 1 µm.[3]

Supplementary Note 1

Comparison of the Electrochemical Performance of the EC-TEM Cell and a Standard H-Type Cell

To determine if the electrochemical data obtained from the EC-TEM cell accurately reproduced the behavior found in the benchtop measurements, we compared the current densities that were obtained after correcting for the surface area of the Cu catalysts. The exposed surface area of Cu ($area_{Cu}$) was estimated from the size and distribution of the as-synthesized cubes in the electron microscopy images. Here, we assume that each cube has 5 exposed facets of similar size, with the sixth one covered in contact with the support. The contribution to the current from the pure glassy carbon plate is negligible compared to the catalysts. For the *ex situ* experiments, SEM images of at least three different locations have been taken on the sample and the number of cubes per μm^2 as well as their average size have been determined. For the *in situ* experiments, the same has been done using the STEM images. The linear sweep voltammetry currents and chronoamperometric traces have been normalized by dividing the measured currents by the total surface area of Cu.

We are aware that such normalization is not ideal, since the total Cu surface area is changing under reaction conditions due to the morphological changes in the catalysts, as shown our *in situ* experiments. Nevertheless, we consider that this approach is a reasonable approximation as it captures the differences that are caused by the initially distinct cube size and loading, which would be otherwise ignored if the support geometrical surface area is used for the normalization.

In addition, we performed further measurements to compare the onset potentials of CO_2RR and Cu dissolution in the holder and in the flask to check that the applied potential in the EC-TEM microfluidic cell can be translated to that found in our standard-type cell. First, we applied a structured cyclic potential recipe to electrodeposit Cu_2O particles on the EC-TEM chips and the glassy carbon plates.[4] Then, these samples were subjected to a series of cyclic voltammetry scans at 25 mV/s in 0.1 M $KHCO_3$ (Supplementary Figure 22). Such an approach is necessary because a sustained oxidative potential quickly drives the complete dissolution of Cu from the carbon surfaces. From these measurements, we determined that the cyclic voltammograms obtained from these specially fabricated reference electrodes are reproducibly shifted by ~-0.4 V from the cyclic voltammograms obtained from the H-type cell. We attribute the measured offset to the unique design of the EC-TEM cell/holder and the longer (fixed) separation between the reference and working electrodes. To mitigate any discrepancy in the applied potential, we adopted an additional calibration step during our experiments to ensure that we are reproducibly applying the right potential. Every experiment was started with two linear sweep voltammograms and then, the profiles were compared to the CO_2RR onset potential to our benchtop reference measurement of a sample with similar loading and size (Supplementary Figure 23 compares LSVs taken on different days). These measurements also indicate that the reference potential remained reasonably stable over long periods of time. In general, we only found relatively small day-to-day shifts (< 50 mV) between different experiments when using the same reference electrode. The day-to-day shifts can be explained by degradation of the KCl solution or the AgCl in the reference electrode over time, both of which needs to be much smaller to fit within the TEM holder.

An example where the initial linear sweep voltammograms are superimposed after correcting for the reference potential offset and normalization over the exposed surface area of Cu for the samples in the two setups is shown in Supplementary Figure 24. It is clear from the profiles that the current densities also compare well against each other. We emphasize here that our experiments have shown that the structural changes in range of -0.9 V_{RHE} to -1.3 V_{RHE} is not strongly potential dependent and only the rate of change is affected by applied potential. Hence, both time-resolved imaging and electrochemical data from both in situ TEM and benchtop setups indicate a robust correspondence between the two systems, allowing us to extrapolate the *in situ* findings and

associate them with the real changes in catalytic performance measured from the identically prepared samples on the bulk electrodes.

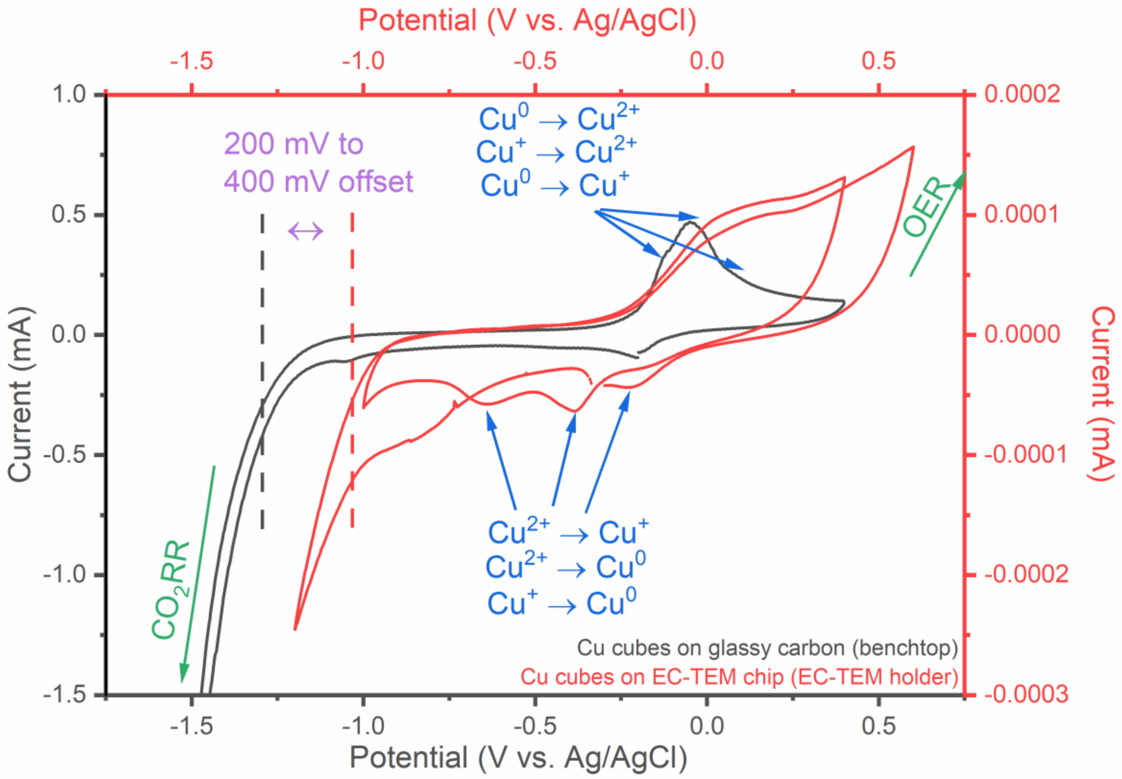

Supplementary Figure 22. Cyclic voltammetry comparison of the benchtop setup vs. the EC-TEM holder electrochemical cell. CVs of Cu_2O cubes on glassy carbon (black) and the carbon electrode of the EC-TEM chip (red) taken at 25 mV/s sweep rate in 0.1 M $KHCO_3$. The potential limits of the cyclic voltammetry have been adjusted to probe the CO_2RR onset and Cu red/ox peak positions. The number of cycles were strongly limited since the cubes dissolve fast at oxidative potentials, as can be observed by the decreasing signal intensity.

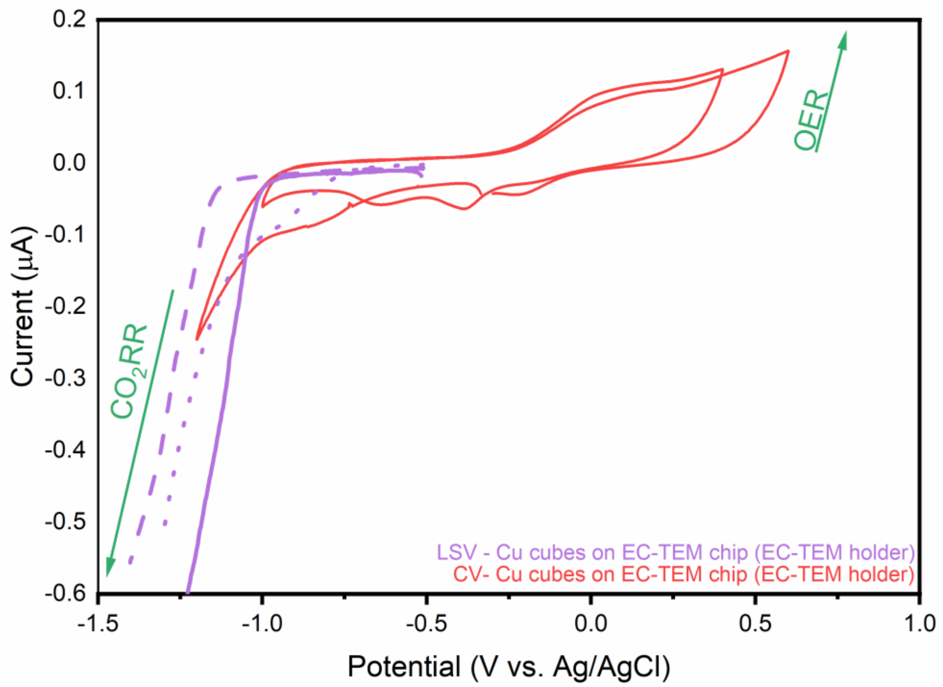

Supplementary Figure 23. Cyclic voltammetry overlaid with linear sweep voltammetry from three different experiments. CVs of Cu_2O cubes on glassy carbon electrode of the EC-TEM chip (red) taken at 25 mV/s sweep rate in 0.1 M $KHCO_3$ and LSVs of 3 different experiment days (purple). The cyclic voltammetry plot is reproduced from Supplementary Figure 22. The potential was corrected by 400 mV prior to day-to-day adjustments.

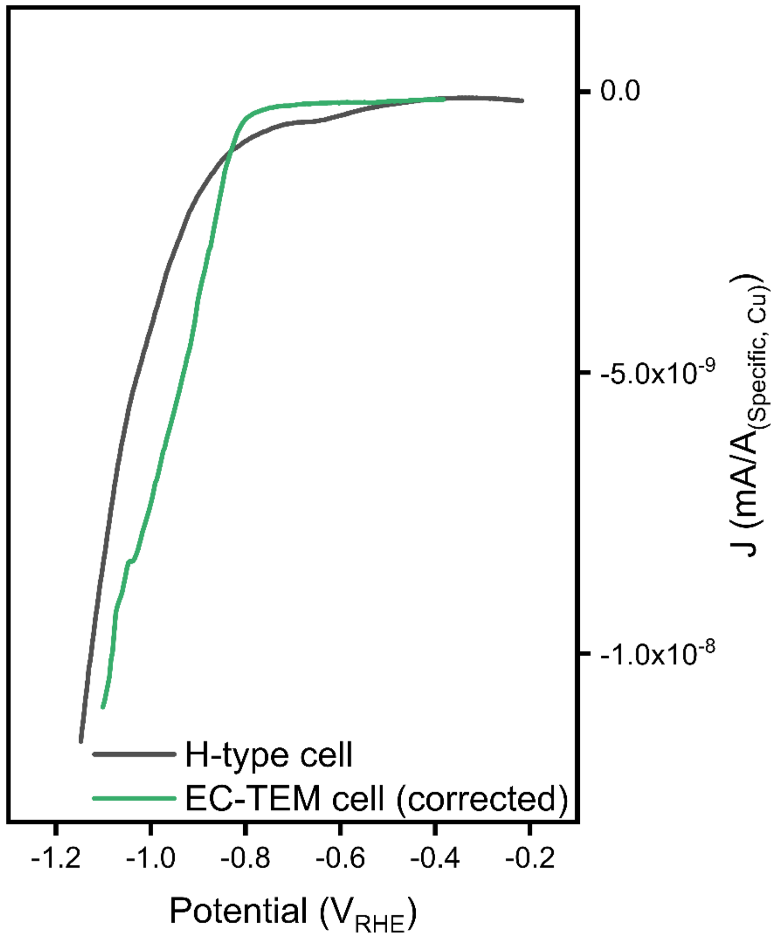

Supplementary Figure 24. Linear sweep voltammogram from *ex situ* and *in situ* experiments. The currents have been normalized over the estimated exposed surface area of Cu on the working electrode, which is a glass carbon plate in the H-type cell and a carbon thin film in the EC-TEM experiments respectively.

Supplementary Note 2

Time-Resolved *Ex Situ* SEM Imaging of Cubes Reacted in a Standard H-Type Cell

To confirm that the EC-TEM observations can be extrapolated to the behavior in a standard electrochemical cell, we further followed *ex situ* the morphological evolution of the Cu_2O cubes deposited on the glassy carbon plates during CO_2RR. The samples were extracted periodically over one hour and investigated by SEM. Supplementary Figure 25 shows a sequence of SEM images from a sample with 170 nm cubes acquired after different CO_2RR times at -0.9 V_{RHE}. Indeed, the SEM images show the restructuring of the cubes and NP re-deposition similar to that observed in the *in situ* experiments. As shown in the histograms in Supplementary Figure 25(b) and 25(c), the cubes in our standard cell also did not change significantly in size and number density during the experiments, whereas the re-deposited NPs increased in size and number.

We mention here that the increase in re-deposited NP size (not resulting from aggregation) and number is artifact of these time-resolved *ex situ* experiments. The repeated removal of the glassy carbon plates from the electrolyte and applied potential, leads to surface re-oxidation and a new cycle of re-deposition when the samples are re-introduced into the reaction environment, as shown in the chronoamperometric plots shown in Supplementary Figure 25(d). This behavior, in turn, results in a perceived increase in re-deposited NPs density as seen in the time-resolved series. In addition, we reiterate that these time-resolved measurements are also not sensitive to catalyst detachment events as shown in Supplementary Figure 5. Therefore, while they can be sufficient for supporting the appearance of structural change during electrochemistry, they should not be used to make any quantitative conclusions regarding the catalyst dynamics.

Lastly, we show in Supplementary Figure 26 the images of the catalysts found on the EC-TEM chips after 1 hour of reaction, which indicates that the morphological changes are consistent with those found on the glassy carbon plates.

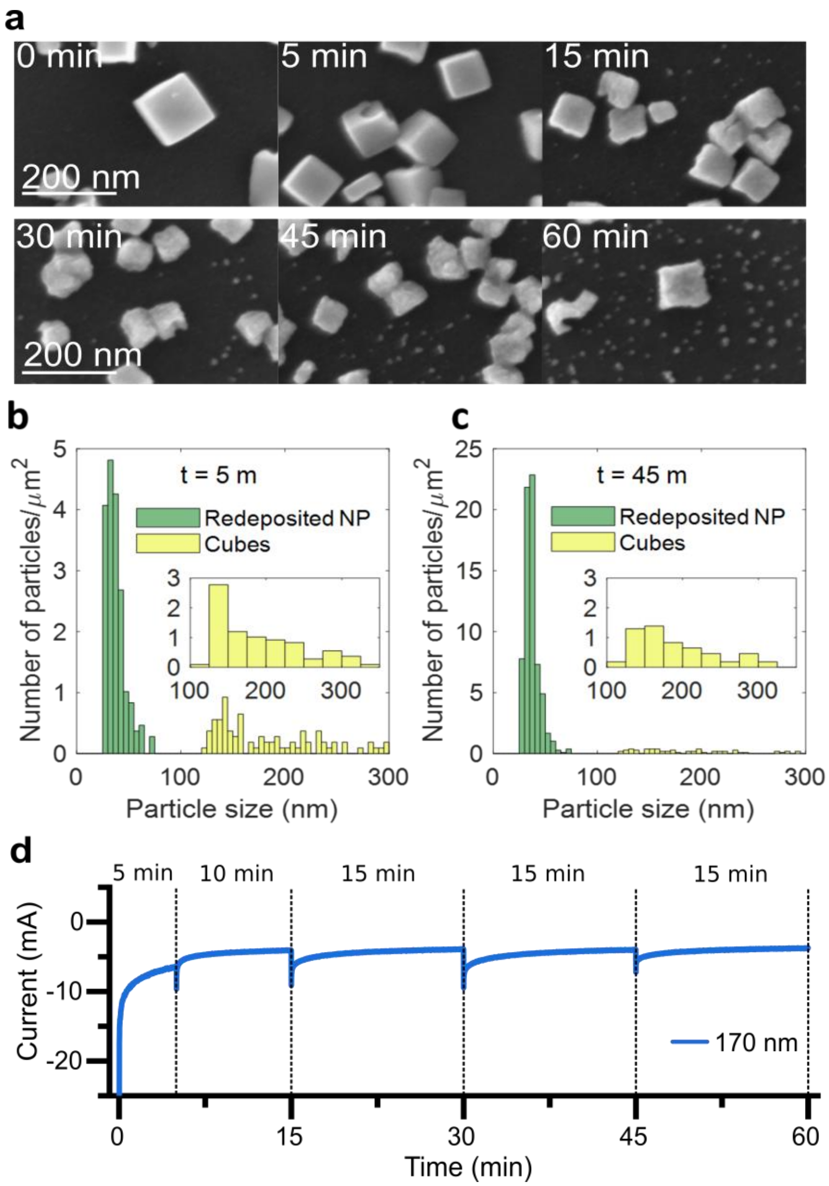

Supplementary Figure 25. Time-resolved *ex situ* SEM of Cu_2O cubes. (a) SEM of 170 nm cubes extracted at different time points of CO_2RR at constant potential (-0.9 V_{RHE}). The reaction was stopped at 5-15 minute intervals, and the glassy carbon support with the specimen were taken out of the electrolyte solution for imaging. Next, it was dried in an N_2 stream and measured by SEM. The histograms compare the size distribution of cubes and re-deposited particles after (b) 5 minutes and (c) 45 minutes. (d) Chronoamperometric trace obtained during these time-resolved experiments. The high initial reductive currents following each re-immersion of the glassy carbon plate suggested that the catalysts re-oxidized when they were removed from the electrolyte and the applied potential. Hence, each re-immersion also introduced a new cycle of dissolution and re-deposition.

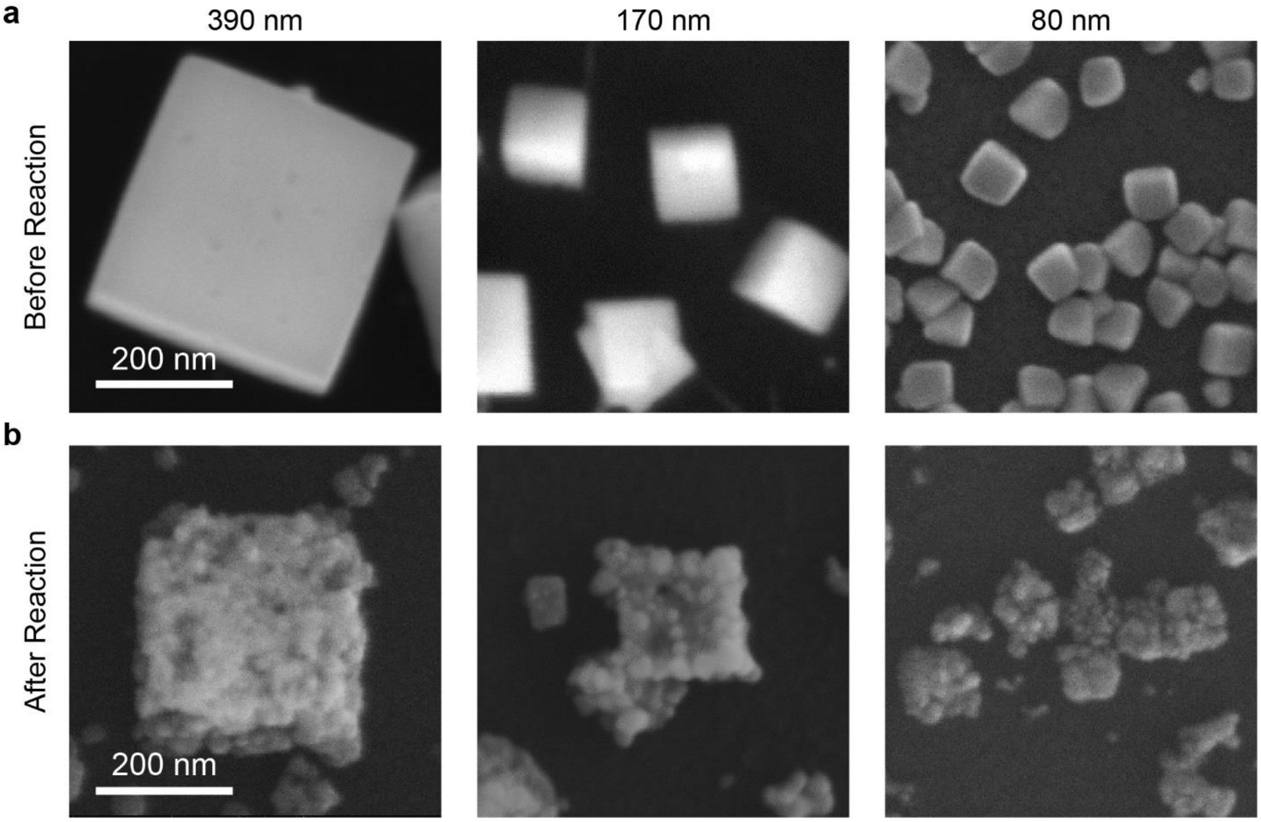

Supplementary Figure 26. Ex situ SEM images of Cu_2O on the EC-TEM chips before and after the CO_2RR reaction. The reaction time was 60 minutes at a potential of -0.9 V_{RHE}. All images are at the same magnification.

Supplementary Note 3

Equations for the Conversion of the Applied Potential to the Reversible Hydrogen Electrode Potential and the Calculation of Faradaic Efficiency of the Reaction Productions

Converting the Applied Potential to the Reversible Hydrogen Electrode Potential

The conversion of the applied potential to the potential of the reversible hydrogen electrode can be done with the Nernst equation:

$$E_{RHE} = E_{Ag/AgCl\,(Sat.)} - \frac{k \bullet T}{n \bullet F}\, Log_{10}(H^{+})$$

E_{RHE}: (converted) potential of the reversible hydrogen electrode

$E_{Ag/AgCl\,(Sat.)}$ = +0.197 V at 25°C (and more positive for lower KCl concentrations)

k: Boltzmann constant: 1.380649×10^{-23} J K^{-1}

T: Temperature

n: number of electrons transferred in the process (here 1)

F: Faraday constant: 96485.33212 C mol^{-1}

$Log_{10}(H^{+})$ = - pH

where $Log_{10}(H^{+})$ is simply the negative of the electrolyte pH value, which is 6.8 for of CO_2 saturated $KHCO_3$.

Formula used for the product analysis

Calculation of Faradaic efficiency of gas products

$$FE\%_{(a,gas)} = \frac{f_{flow} \bullet c_{gas} \bullet n_a \bullet F}{I_{total} \bullet V_m} \bullet 100$$

Calculation of Faradaic efficiency of liquid products

$$FE\%_{(a,liquid)} = \frac{c_{liquid} \bullet V \bullet n_a \bullet F}{Q_{total}} \bullet 100$$

Calculation of partial current density

$$i_a = \frac{FE\%_a}{100} \bullet \frac{i_{total}}{A_{geo.}}$$

f_{flow}: flow rate mL min^{-1}

c_{gas}: concentration gas in mol L^{-1}

c_{liquid}: *accumulated* concentration liquid in mol L^{-1}

V_m: molar volume of an ideal gas at 1 atm in mL mol^{-1}

V: volume of anolyte in the cell in L

Q_{total}: total charge (dI/dt) in C

A_{geo}: geometrical surface area in cm^2

n_a: number of transferred electrons for the respective product "a"

F: Faradaic constant, 96485 C mol^{-1}

i_{total}: total electrolysis current in A

i_a: partial current density for the respective product "a" in A

Supplementary Note 4

Electron Beam-Induced Effects in *In Situ* Experiments

Electron beam-related effects are always a concern for liquid cell TEM experiments, and so we have adopted a low electron dose imaging protocol to minimize these artifacts. In our previous work,[5] we determined the electron flux limit for observing noticeable beam-induced effects in our TEM to be ~7 $e^{-}Å^{-2}\ s^{-1}$ and we stayed under this threshold flux in the current experiments. In Supplementary Figure 27, we present another control experiment where the electron beam was blanked until only after potential application. While it was unfortunate that a bubble formed during the potential sweep, it was clear that we could see the fragmentation of the cubes and the formation of re-deposited NPs. Supplementary Figure 28 further shows the after-reaction morphologies acquired after the *in situ* experiments from the areas not exposed to the electron beam. The cubes located in the two areas do not exhibit significant differences in morphology, indicating that those extended imaging did not significantly alter the cubes. Hence, we can conclude that we were able to avoid significant electron beam-induced artifacts with our low electron dose protocol, even with extended imaging times of about an hour.

There appears, however, to be subtle effects caused by the extended electron beam exposure since there seems to be more secondary NPs in the imaged area as compared to non-imaged areas (Supplementary Figure 29). Nevertheless, this effect does not affect the conclusions of our work the morphologies are similar between the two areas and are also consistent with the behavior found in our *ex situ* SEM experiments described in Supplementary Note 2.

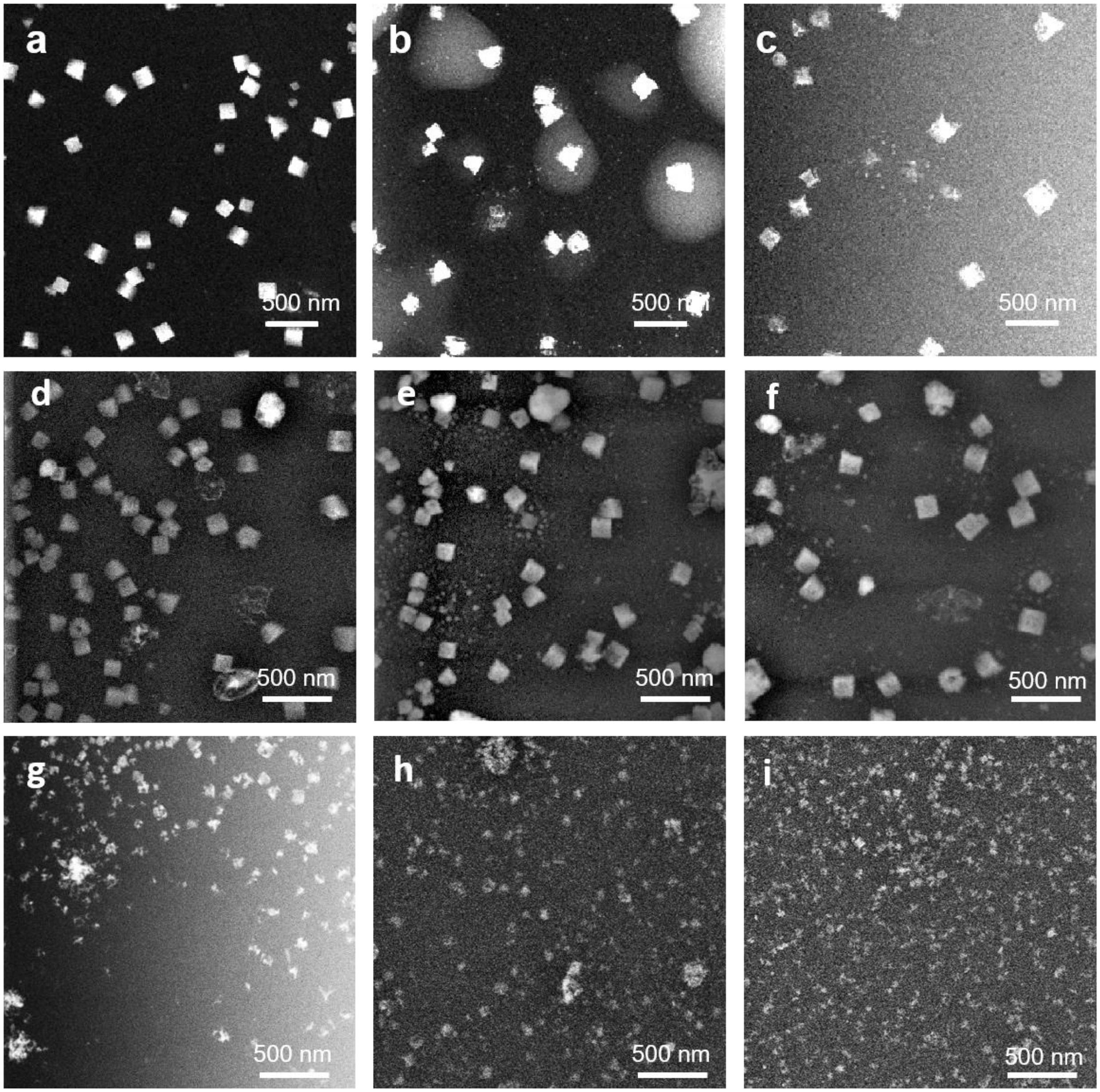

Supplementary Figure 27. Control experiments where the beam was blanked during potential application. (a) *Ex situ* STEM image of the sample before it was loaded into the EC-TEM holder showing that there are no re-deposited NPs. (b)-(c) *In situ* STEM images acquired after a potential sweep to -1.1 V_{RHE} with the holder in the TEM, but while the electron beam was blanked. Although a bubble formed during the potential sweep, it is clear that there are new re-deposited NPs on the working electrode surface. The lighter areas indicate residual electrolyte droplets. Similar experiments with (d-e) more densely loaded cubes and (g-i) smaller starting cubes.

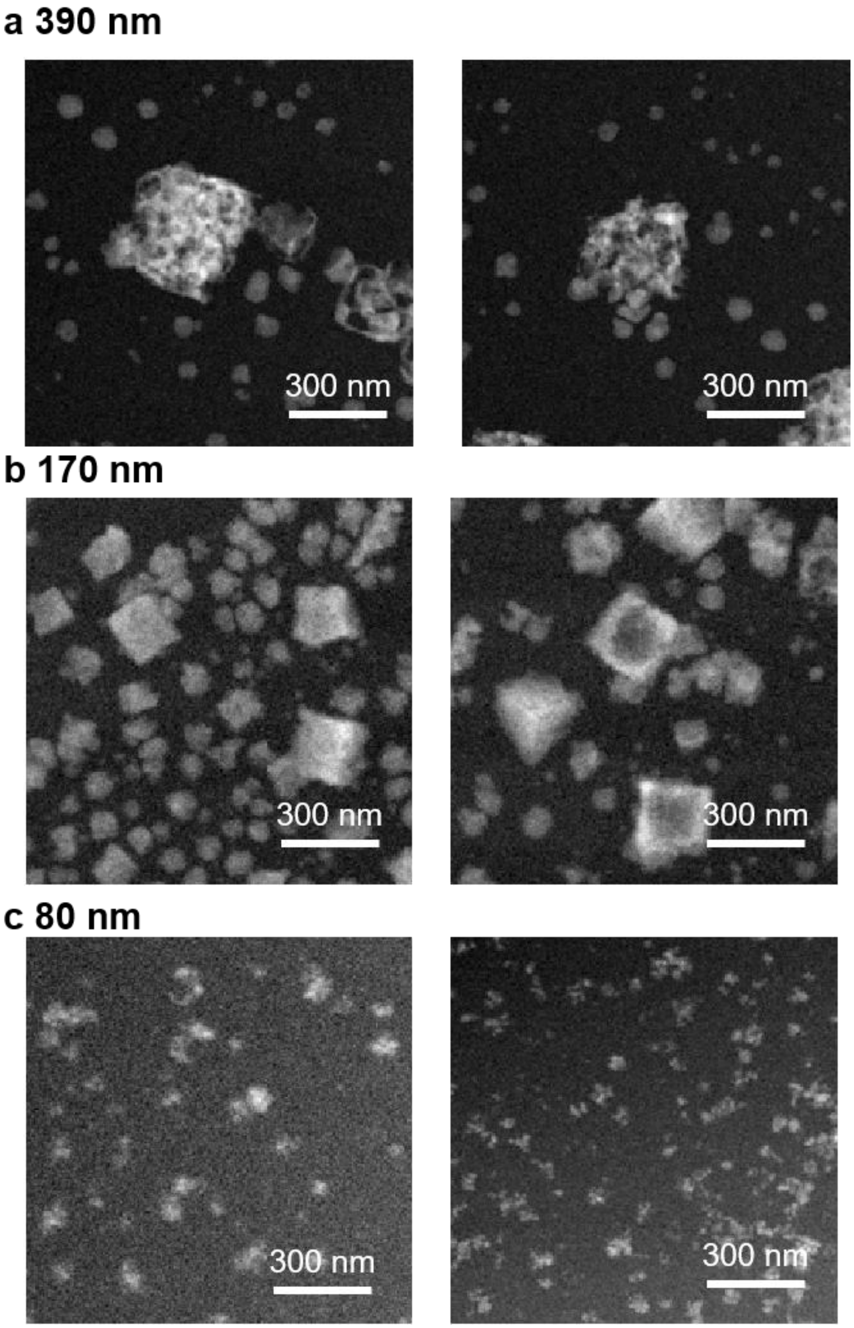

Supplementary Figure 28. ***In situ*** **STEM images acquired after reaction from areas that were not imaged by the electron beam for (a) 390 nm, (b) 170 nm, and (c) 80 nm of cubes.**

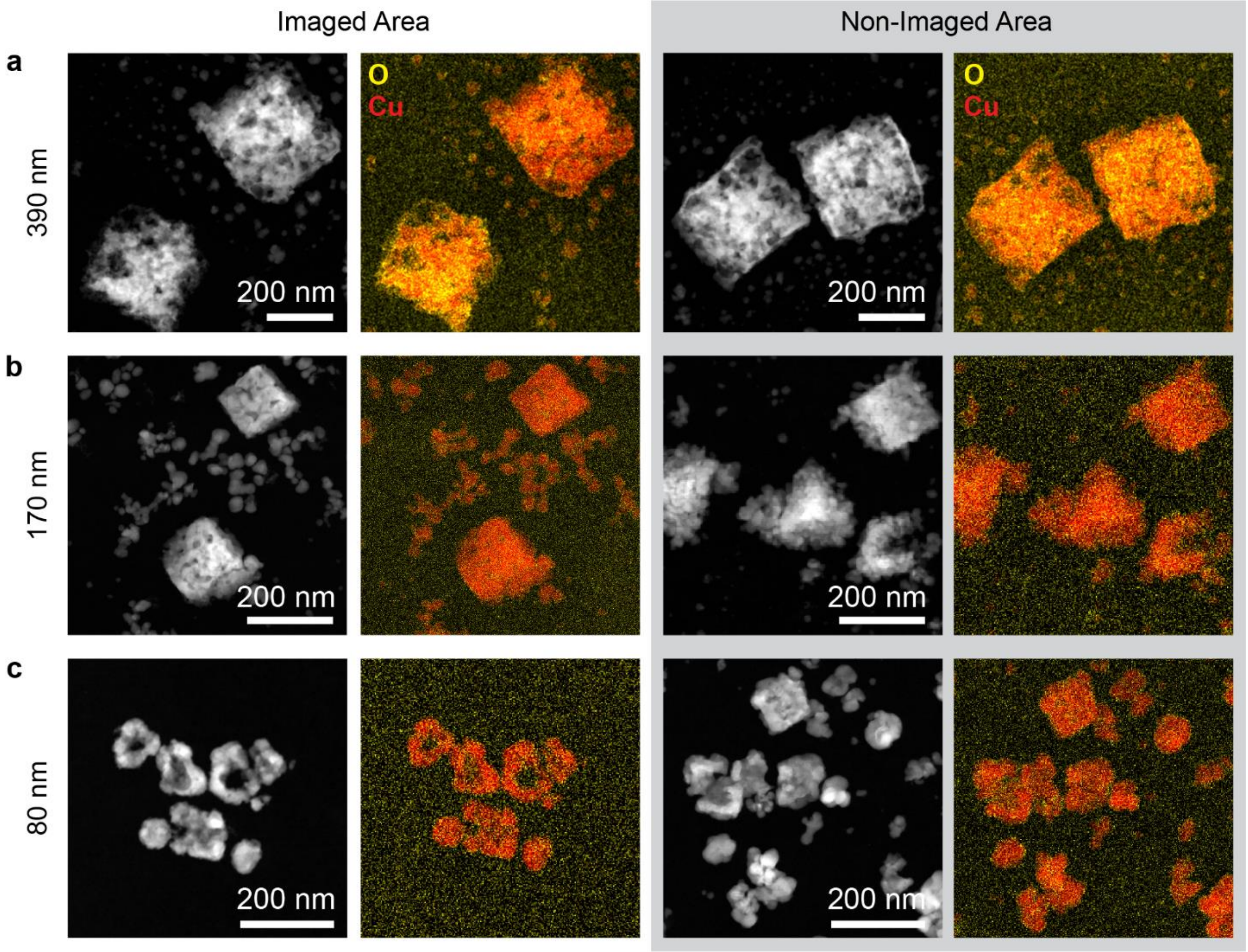

Supplementary Figure 29. ***Ex situ*** **STEM-EDX images of areas imaged by the electron beam and those that were not.** The pairs of STEM images and EDX maps compare Cu_2O cubes found on the areas of the working electrode that were imaged during the *in situ* experiment and cubes found on areas that were not imaged. Images of each set are at the same magnification. The initial cube sizes were (a) 390 nm, (b) 170 nm, and (c) 80 nm.

File Name: Supplementary Movie 1

Description: EC-TEM movie illustrating the structural change of the 170 nm Cu_2O cubes during the 1st linear sweep voltammetry (LSV) from -0.3 to -1.1 V_{RHE}. It shows the immediate deposition of the small nanoparticles with the applied potential. The plot on the right shows the electrochemical response as a function of the applied potential. The recording rate of the movie was 1 frame per second. The movie playback rate is in real time. The electron flux was 1.7 $e^- Å^{-2} s^{-1}$.

File Name: Supplementary Movie 2

Description: EC-TEM movie displaying the structural changes in the Cu_2O cubes during the 2nd LSV from -0.3 to -1.0 V_{RHE} in a different experiment. The plot on the right shows the electrochemical response as a function of the applied potential. The redeposited particles in the first frames were formed during the 1st LSV. The image sequence was first drift-corrected and then, two frames were averaged to create one frame of the movie. The movie playback rate is in real time. The electron flux was 1.7 $e^- Å^{-2} s^{-1}$.

File Name: Supplementary Movie 3

Description: EC-TEM movie depicting the structural change of the Cu_2O cubes in movie 1 in the subsequent chronoamperometry at -1.1 V_{RHE} in 0.1 M $KHCO_3$ for 9 minutes. The movie is cut at 6 min 30 seconds when bubble formed. The recording rate of the movie was 1 frame per second. 6 frames were averaged to create one frame of the movie. The movie playback rate is ×30 times real time. The electron flux was 1.7 $e^- Å^{-2} s^{-1}$.

File Name: Supplementary Movie 4

Description: EC-TEM movie describing the motion of particles driven by bubble formation and their removal process during the chronoamperometry at -1.1 $V_{RHE.}$ The movie play back rate is ×5 times real time. The electron flux was 0.11 $e^- Å^{-2} s^{-1}$.

File Name: Supplementary Movie 5

Description: EC-TEM movie describing the structural changes in the 390 nm Cu_2O cubes during 1 h of chronoamperometry at -0.9 V_{RHE} in 0.1 M $KHCO_3$. The recording rate of the movie was 1

frame per second. 5 frames were averaged to create one frame of the movie. The movie playback rate is ×100 times real time. The electron flux was 3.5 e^{-} $Å^{-2}$ s^{-1}.

File Name: Supplementary Movie 6

Description: EC-TEM movie describing the structural changes in the 170 nm Cu_2O cubes during 1 h of chronoamperometry at -0.9 V_{RHE} in 0.1 M $KHCO_3$. The recording rate of the movie was 1 frame per second. 5 frames were averaged to create one frame of the movie. The movie playback rate is ×100 times real time. The electron flux was 3.5 e^{-} $Å^{-2}$ s^{-1}.

File Name: Supplementary Movie 7

Description: EC-TEM movie describing the structural changes in the 80 nm Cu_2O cubes during 50 min of chronoamperometry at -0.9 V_{RHE} in 0.1 M $KHCO_3$ from an area that has mostly cubes. The recording rate of the movie was 1 frame per second. 5 frames were averaged to create one frame of the movie. The movie playback rate is ×100 times real time. The electron flux was 3.5 e^{-} $Å^{-2}$ s^{-1}.

File Name: Supplementary Movie 8

Description: EC-TEM movie describing the structural changes in the 80 nm Cu_2O cubes during 45 min of chronoamperometry at -0.9 V_{RHE} in 0.1 M $KHCO_3$ from an area that has mostly partial cube fragments and re-deposited NPs. The recording rate of the movie was 1 frame per second. 5 frames were averaged to create one frame of the movie. The movie playback rate is ×100 times real time. The electron flux was 3.5 e^{-} $Å^{-2}$ s^{-1}.

File Name: Supplementary Movie 9

EC-TEM movie describing the structural changes in the 390 nm Cu_2O cubes during 25 min of chronoamperometry at -0.9 V_{RHE} in 0.1 M $KHCO_3$. The recording rate of the movie was 1 frame per second. 10 frames were averaged to create one frame of the movie. The movie playback rate is ×200 times real time. The electron flux was 1.7 e^{-} $Å^{-2}$ s^{-1}.

File Name: Supplementary Movie 10

EC-TEM movie describing the structural changes in the 170 nm Cu_2O cubes during 25 min of chronoamperometry at -0.9 V_{RHE} in 0.1 M $KHCO_3$. The recording rate of the movie was 1 frame

per second. 10 frames were averaged to create one frame of the movie. The movie playback rate is ×200 times real time. The electron flux was 1.7 e^{-} $Å^{-2}$ s^{-1}.

File Name: Supplementary Movie 11

EC-TEM movie describing the structural changes in the 80 nm Cu_2O cubes during 25 min of chronoamperometry at -0.9 V_{RHE} in 0.1 M $KHCO_3$. The recording rate of the movie was 1 frame per second. 10 frames were averaged to create one frame of the movie. The movie playback rate is ×200 times real time. The electron flux was 1.7 e^{-} $Å^{-2}$ s^{-1}.

File Name: Supplementary Movie 12

Description: EC-TEM movie describing the structural changes in the 390 nm Cu_2O cubes with higher loading than Movie 5 during 1 h of chronoamperometry at -0.9 V_{RHE} in 0.1 M $KHCO_3$. The recording rate of the movie was 1 frame per second. 10 frames were averaged to create one frame of the movie. The movie playback rate is ×200 times real time. The electron flux was 1.7 e^{-} $Å^{-2}$ s^{-1}.

File Name: Supplementary Movie 13

Description: EC-TEM movie describing the structural changes in the 170 nm Cu_2O cubes with lower loading than Movie 6 during 1 h of chronoamperometry at -0.9 V_{RHE} in 0.1 M $KHCO_3$. The recording rate of the movie was 1 frame per second. 10 frames were averaged to create one frame of the movie. The movie playback rate is ×200 times real time. The electron flux was 1.7 e^{-} $Å^{-2}$ s^{-1}.

File Name: Supplementary Movie 14

Description: EC-TEM Movie describing the structural changes in the 30 nm Cu_2O cubes synthesized by colloidal chemistry during 30 min of chronoamperometry at -0.9 V_{RHE} in 0.1 M $KHCO_3$. The recording rate of the movie was 1 frame per second. 5 frames were averaged to create one frame of the movie. The movie playback rate is ×100 times real time. The electron flux was 13 e^{-} $Å^{-2}$ s^{-1}. Due to the small size of these Cu cubes, a higher electron flux had to be used for in these experiments. Nevertheless, comparisons between electron irradiated and non-irradiated areas of the sample reveal the similar morphologies of the catalysts after reaction.

References

1. Beker, A. F. *et al.* In situ electrochemistry inside a TEM with controlled mass transport. *Nanoscale* **12**, 22192–22201 (2020).
2. Grosse, P. *et al.* Dynamic Changes in the Structure, Chemical State and Catalytic Selectivity of Cu Nanocubes during CO_2 Electroreduction: Size and Support Effects. *Angew. Chemie - Int. Ed.* **57**, 6192–6197 (2018).
3. Holtz, M. E., Yu, Y., Gao, J., Abruña, H. D. & Muller, D. A. In Situ Electron Energy Loss Spectroscopy in Liquids. *Microsc. Microanal.* **19**, 1027–1035 (2013).
4. Grosse, P., Yoon, A., Rettenmaier, C., Chee, S. W. & Cuenya, B. R. Growth Dynamics and Processes Governing the Stability of Electrodeposited Size-Controlled Cubic Cu Catalysts. *J. Phys. Chem. C* **124**, 26908–26915 (2020).
5. Arán-Ais, R. M. *et al.* Imaging electrochemically synthesized Cu2O cubes and their morphological evolution under conditions relevant to CO_2 electroreduction. *Nat. Commun.* **11**, 3489 (2020).
