# Supplementary material for: Dynamic transformation of cubic copper catalysts during CO2 electroreduction and its impact on catalytic selectivity
Source: Nat Commun. 2021 Nov 18;12:6736. doi: 10.1038/s41467-021-26743-5 (PMC8602378; doi:10.1038/s41467-021-26743-5)
Supplement: Supplementary file 2 — Supplementary Movie legends [file 41467_2021_26743_MOESM2_ESM.docx]

**File Name: Supplementary Movie 1**

Description: EC-TEM movie illustrating the structural change of the 170 nm Cu_2_O cubes during the 1^st^ linear sweep voltammetry (LSV) from -0.3 to -1.1 V_RHE_. It shows the immediate deposition of the small nanoparticles with the applied potential. The plot on the right shows the electrochemical response as a function of the applied potential. The recording rate of the movie was 1 frame per second. The movie playback rate is in real time. The electron flux was 1.7 e^-^ Å^‑2^ s^‑1^.

**File Name: Supplementary Movie 2**

Description: EC-TEM movie displaying the structural changes in the Cu_2_O cubes during the 2^nd^ LSV from -0.3 to -1.0 V_RHE_ in a different experiment. The plot on the right shows the electrochemical response as a function of the applied potential. The redeposited particles in the first frames were formed during the 1^st^ LSV. The image sequence was first drift-corrected and then, two frames were averaged to create one frame of the movie. The movie playback rate is in real time. The electron flux was 1.7 e^-^ Å^-2^ s^-1^.

**File Name: Supplementary Movie 3**

Description: EC-TEM movie depicting the structural change of the Cu_2_O cubes in movie 1 in the subsequent chronoamperometry at -1.1 V_RHE_ in 0.1 M KHCO_3_ for 9 minutes. The movie is cut at 6 min 30 seconds when bubble formed. The recording rate of the movie was 1 frame per second. 6 frames were averaged to create one frame of the movie. The movie playback rate is ×30 times real time. The electron flux was 1.7 e^-^ Å^-2^ s^-1^.

**File Name: Supplementary Movie 4**

Description: EC-TEM movie describing the motion of particles driven by bubble formation and their removal process during the chronoamperometry at -1.1 V_RHE._ The movie play back rate is ×5 times real time. The electron flux was 0.11 e^-^ Å^-2^ s^-1^.

**File Name: Supplementary Movie 5**

Description: EC-TEM movie describing the structural changes in the 390 nm Cu_2_O cubes during 1 h of chronoamperometry at -0.9 V_RHE_ in 0.1 M KHCO_3_. The recording rate of the movie was 1 frame per second. 5 frames were averaged to create one frame of the movie. The movie playback rate is ×100 times real time. The electron flux was 3.5 e^-^ Å^-2^ s^-1^.

**File Name: Supplementary Movie 6**

Description: EC-TEM movie describing the structural changes in the 170 nm Cu_2_O cubes during 1 h of chronoamperometry at -0.9 V_RHE_ in 0.1 M KHCO_3_. The recording rate of the movie was 1 frame per second. 5 frames were averaged to create one frame of the movie. The movie playback rate is ×100 times real time. The electron flux was 3.5 e^-^ Å^-2^ s^-1^.

**File Name: Supplementary Movie 7**

Description: EC-TEM movie describing the structural changes in the 80 nm Cu_2_O cubes during 50 min of chronoamperometry at -0.9 V_RHE_ in 0.1 M KHCO_3_ from an area that has mostly cubes. The recording rate of the movie was 1 frame per second. 5 frames were averaged to create one frame of the movie. The movie playback rate is ×100 times real time. The electron flux was 3.5 e^-^ Å^-2^ s^‑1^.

**File Name: Supplementary Movie 8**

Description: EC-TEM movie describing the structural changes in the 80 nm Cu_2_O cubes during 45 min of chronoamperometry at -0.9 V_RHE_ in 0.1 M KHCO_3_ from an area that has mostly partial cube fragments and re-deposited NPs. The recording rate of the movie was 1 frame per second. 5 frames were averaged to create one frame of the movie. The movie playback rate is ×100 times real time. The electron flux was 3.5 e^-^ Å^-2^ s^‑1^.

**File Name: Supplementary Movie 9**

EC-TEM movie describing the structural changes in the 390 nm Cu_2_O cubes during 25 min of chronoamperometry at -0.9 V_RHE_ in 0.1 M KHCO_3_. The recording rate of the movie was 1 frame per second. 10 frames were averaged to create one frame of the movie. The movie playback rate is ×200 times real time. The electron flux was 1.7 e^-^ Å^-2^ s^-1^.

**File Name: Supplementary Movie 10**

EC-TEM movie describing the structural changes in the 170 nm Cu_2_O cubes during 25 min of chronoamperometry at -0.9 V_RHE_ in 0.1 M KHCO_3_. The recording rate of the movie was 1 frame per second. 10 frames were averaged to create one frame of the movie. The movie playback rate is ×200 times real time. The electron flux was 1.7 e^-^ Å^-2^ s^-1^.

**File Name: Supplementary Movie 11**

EC-TEM movie describing the structural changes in the 80 nm Cu_2_O cubes during 25 min of chronoamperometry at -0.9 V_RHE_ in 0.1 M KHCO_3_. The recording rate of the movie was 1 frame per second. 10 frames were averaged to create one frame of the movie. The movie playback rate is ×200 times real time. The electron flux was 1.7 e^-^ Å^-2^ s^-1^.

**File Name: Supplementary Movie 12**

Description: EC-TEM movie describing the structural changes in the 390 nm Cu_2_O cubes with higher loading than Movie 5 during 1 h of chronoamperometry at -0.9 V_RHE_ in 0.1 M KHCO_3_. The recording rate of the movie was 1 frame per second. 10 frames were averaged to create one frame of the movie. The movie playback rate is ×200 times real time. The electron flux was 1.7 e^-^ Å^-2^ s^‑1^.

**File Name: Supplementary Movie 13**

Description: EC-TEM movie describing the structural changes in the 170 nm Cu_2_O cubes with lower loading than Movie 6 during 1 h of chronoamperometry at -0.9 V_RHE_ in 0.1 M KHCO_3_. The recording rate of the movie was 1 frame per second. 10 frames were averaged to create one frame of the movie. The movie playback rate is ×200 times real time. The electron flux was 1.7 e^-^ Å^-2^ s^‑1^.

**File Name: Supplementary Movie 14**

Description: EC-TEM Movie describing the structural changes in the 30 nm Cu_2_O cubes synthesized by colloidal chemistry during 30 min of chronoamperometry at -0.9 V_RHE_ in 0.1 M KHCO_3_. The recording rate of the movie was 1 frame per second. 5 frames were averaged to create one frame of the movie. The movie playback rate is ×100 times real time. The electron flux was 13 e^-^ Å^-2^ s^-1^. Due to the small size of these Cu cubes, a higher electron flux had to be used for in these experiments. Nevertheless, comparisons between electron irradiated and non-irradiated areas of the sample reveal the similar morphologies of the catalysts after reaction.
